# Chronic inflammation with microglia senescence at basal forebrain: impact on cholinergic deficit in Alzheimer’s brain haemodynamics

**DOI:** 10.1093/braincomms/fcae204

**Published:** 2024-06-12

**Authors:** Dong Liu, Shih Chang Hsueh, David Tweedie, Nate Price, Elliot Glotfelty, Daniela Lecca, Richard Telljohann, Rafael deCabo, Barry J Hoffer, Nigel H Greig

**Affiliations:** Drug Design & Development Section, Translational Gerontology Branch, Intramural Research Program, National Institute on Aging, National Institutes of Health, Baltimore, MD 21224, USA; Drug Design & Development Section, Translational Gerontology Branch, Intramural Research Program, National Institute on Aging, National Institutes of Health, Baltimore, MD 21224, USA; Department of Pediatrics, Columbia University Irving Medical Center, Columbia University Vagelos Physicians & Surgeons College of Medicine, New York City, NY 10032, USA; Drug Design & Development Section, Translational Gerontology Branch, Intramural Research Program, National Institute on Aging, National Institutes of Health, Baltimore, MD 21224, USA; Experimental Gerontology Section, Translational Gerontology Branch, Intramural Research Program, National Institute on Aging, National Institutes of Health, Baltimore, MD 21224, USA; Drug Design & Development Section, Translational Gerontology Branch, Intramural Research Program, National Institute on Aging, National Institutes of Health, Baltimore, MD 21224, USA; Department of Neuroscience, Karolinska Institute, Stockholm 17177, Sweden; Cellular Stress and Inflammation Section, Intramural Research Program, National Institute on Drug Abuse, Baltimore, MD 21224, USA; Drug Design & Development Section, Translational Gerontology Branch, Intramural Research Program, National Institute on Aging, National Institutes of Health, Baltimore, MD 21224, USA; Shock, Trauma & Anesthesiology Research Center, University of Maryland, Baltimore, MD 21201, USA; Laboratory of Cardiovascular Science, Intramural Research Program, National Institute on Aging, National Institutes of Health, Baltimore, MD 21224, USA; Department of Pediatrics, Columbia University Irving Medical Center, Columbia University Vagelos Physicians & Surgeons College of Medicine, New York City, NY 10032, USA; Department of Neurosurgery, Case Western Reserve University School of Medicine, University Hospitals, Cleveland, OH 44106, USA; Drug Design & Development Section, Translational Gerontology Branch, Intramural Research Program, National Institute on Aging, National Institutes of Health, Baltimore, MD 21224, USA

**Keywords:** CBF haemodynamics to ACh, Alzheimer’s disease, basal forebrain cholinergic neuron, microglia SASP, chronic neuroinflammation

## Abstract

Cholinergic innervation in the brain is involved in modulating neurovascular function including cerebral blood flow haemodynamics in response to neuronal activity. Cholinergic deficit is associated with pathophysiology in Alzheimer’s disease, albeit the aetiology remains to be clarified. In the current study, neocortex cerebral blood flow response to acetylcholine was evaluated by Laser-Doppler Flowmetry (LDF) in 3xTgAD Alzheimer’s disease model) and wild-type mice of two age groups. The peak of cerebral blood flow to acetylcholine (i.v.) from baseline levels (% ΔrCBF) was higher in young 3xTgAD versus in wild-type mice (48.35; 95% CI:27.03–69.67 versus 22.70; CI:15.5–29.91, *P* < 0.05); this was reversed in old 3xTgAD mice (21.44; CI:2.52–40.35 versus 23.25; CI:23.25–39). Choline acetyltransferase protein was reduced in neocortex, while cerebrovascular reactivity to acetylcholine was preserved in young 3×TgAD mice. This suggests endogenous acetylcholine deficit and possible cholinergic denervation from selected cholinergic nuclei within the basal forebrain. The early deposition of tauopathy moieties (mutant hTau and pTau181) and its coincidence in cholinergic cell clusters (occasionaly), were observed at the basal forebrain of 3xTgAD mice including substantia innominate, nucleus Basalis of Meynert and nucleus of horizontal limb diagonal band of Broca. A prominent feature was microglia interacting tauopathy and demonstrated a variety of morphology changes particularly when located in proximity to tauopathy. The microglia ramified phenotype was reduced as evaluated by the ramification index and Fractal analysis. Increased microglia senescence, identified as SASP (senescence-associated secretory phenotype), was colocalization with p16^Ink4ɑ^, a marker of irreversible cell-cycle arrest in old 3xTgAD versus wild-type mice (*P* = 0.001). The p16^Ink4ɑ^ was also observed in neuronal cells bearing tauopathy within the basal forebrain of 3xTgAD mice. TNF-ɑ, the pro-inflammatory cytokine elevated persistently in microglia (Pearson’s correlation coefficient = 0.62) and the loss of cholinergic cells in vulnerable basal forebrain environment, was indicated by image analysis in 3xTgAD mice, which linked to the cholinergic deficits in neocortex rCBF haemodynamics. Our study revealed the early change of CBF haemodynamics to acetylcholine in 3xTgAD model. As a major effector of brain innate immune activation, microglia SASP with age-related disease progression is indicative of immune cell senescence, which contributes to chronic inflammation and cholinergic deficits at the basal forebrain. Targeting neuroinflammation and senescence may mitigate cholinergic pathophysiology in Alzheimer’s disease.

## Teaser

Liu et al. report that cholinergic innervation modulates neurovascular function. The cerebral blood flow haemodynamics to acetylcholine challenge was altered in neocortex of Alzheimer’s mouse model suggesting cholinergic deficiency. Tauopathy and microglia senescence at the basal forebrain associated with chronic inflammation, which contributes to cholinergic loss and aberrant haemodynamics.

## Graphical Abstract

**Graphical Abstract:**
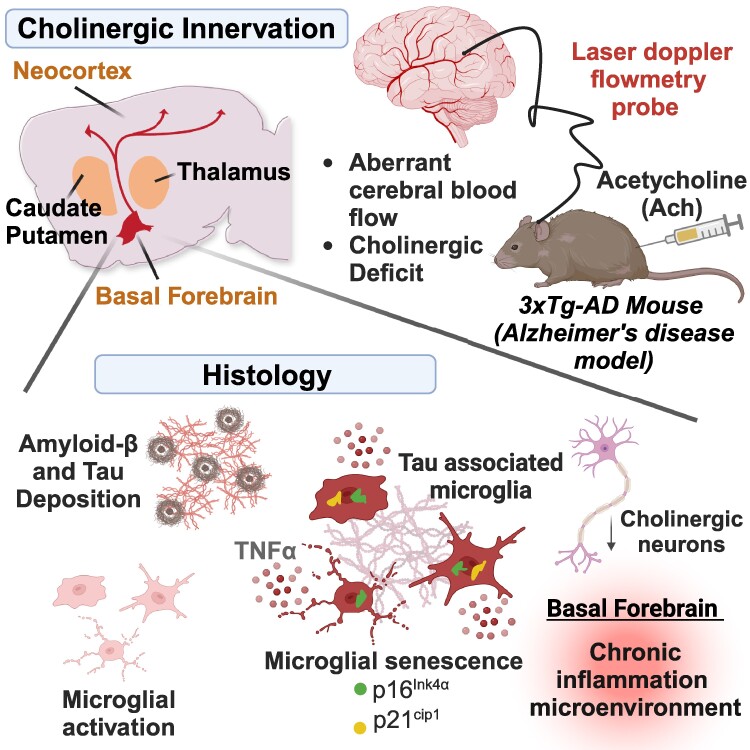

## Introduction

The brain's cholinergic system plays an important role in both the pathophysiology and treatment of Alzheimer’s disease (AD).^1-3^ Cholinergic innervation is distributed ubiquitously throughout the brain and participates in attention, learning, working memory, and neuropsychiatric actions.^4^ Previous studies discovered that drugs with anticholinergic activity, such as acetylcholine (ACh) muscarinic receptor antagonists, caused short-term memory impairment and increased the risk of dementia in older adults.^5,6^ In contrast, acetylcholinesterase inhibitors (AChEIs), which reduce ACh hydrolysis and thereby elevate ACh within the synaptic cleft, enhance memory acquisition. AChEIs remain important for symptomatic pharmacotherapy in the present treatment of AD patients. Previous studies have also reported a selective loss of cholinergic neurons within the basal forebrain (BF) and cholinergic denervation in human AD and in animal models.^7-9^ The aetiology of cholinergic deficits in AD remains to be fully clarified.

Brain cholinergic innervation exerts an impact on cerebral neurovascular function. ACh is the major neurotransmitter for the brain's cholinergic system and is additionally a potent vasodilator that can modulate cerebral neurovascular functions, including cholinergic tone, neurovascular coupling and regional cerebral blood flow (rCBF) haemodynamics.^10-12^ ACh binds to muscarinic ACh receptors (mAChRs) in vascular endothelial cells, induces intrinsic intracellular [Ca^2+^]_i_ responses, nitric oxide (NO) release and vasodilation.^13-15^ Choline, the precursor of ACh, additionally, acts as a selective agonist on the α7-nicotinic acetylcholine receptor in sympathetic nerves to cause vasodilatation.^16^ Central cholinergic stimulation enhances cholinergic activity and CBF in AD patients.^17,18^ The coincidence of early cholinergic deficits and cerebrovascular alterations in AD cases has elicited attention in relation to the cerebrovascular role of cholinergic innervation in AD pathogenesis and pathophysiology.^16,19-21^

The objective of this study is to test the hypothesis that the cholinergic deficits evident in AD potentially impair neurovascular coupling and CBF haemodynamics in the AD brain^10,22,23^; this, in turn, leads to chronic cerebral hypoperfusion, exacerbated AD pathology and cognitive decline.^12,24^ Studies have reported that AChEIs can improve cerebrovascular perfusion through stimulation of the intrinsic cholinergic cerebrovascular innervation in AD and other dementias.^25-27^ In contrast, anticholinergic medications used in older adults with cardiovascular disease were associated with an increased risk of cognitive impairment and dementia.^28^ A deeper understanding of the aetiology of cholinergic deficits in AD could potentially increase success in the design and development of effective disease-modifying interventions for AD, the most common age-related neurodegenerative disease leading to dementia.

Our previous study and those of others indicate that the rCBF haemodynamics in response to neuronal activity (referred to as ‘neurovascular coupling’) is essential for cognition and is altered early in AD patients and rodent AD models.^22,29,30^ To evaluate the cholinergic impacts on cerebrovascular function in AD, the rCBF haemodynamic responses to ACh were investigated here by LDF assessment as an indicator of neurovascular coupling, in middle-aged adult (6–14 months) and old (16–24 months and over) Alzheimer’s (3xTgAD) and age-matched wild-type (WT) mice. The haemodynamics of rCBF response to ACh (i.v.) at neocortex was found to be higher in young AD versus age-matched WT mice, suggesting alterations in endogenous ACh level and/or cholinergic innervation derived from cholinergic nuclei in the BF of AD mice.^31^ Further study revealed an early deposition of tauopathy (human mutant tau P301L and phosphorylated tau at Thr181) within BF of 3xTgAD mice, which were also localized with selected choline acetyltransferase (ChAT)^+^ cell clusters within the SI (substantia innominata), nBM (nucleus Basalis of Meynert) and HDB (nucleus of horizontal limb diagonal band of Broca). Moreover, reactive Iba1^+^ microglia interacted with tauopathy and showed increased dystrophic/senescent phenotypes and p16^Ink4ɑ^ expression (a cell senescent marker of irreversible cell-cycle arrest). The p16^Ink4ɑ^ expression also was observed in neuronal-like cells bearing tauopathy within the BF of AD mice. Persistent elevation of TNF-ɑ, the pro-inflammatory cytokine, was found in Iba1^+^ microglia and the microenvironment of BF even in very elderly AD mice, indicating innate immunity activation and immune cell senescence. This likely induced a chronic neuroinflammatory microenvironment and contributed to the cholinergic loss and pathophysiology in AD brains.

## Rationale

The brain cholinergic innervation modulates neurovascular function for cognition, includes cerebral blood flow (rCBF) haemodynamics in response to neuronal activity. The aetiology of the cholinergic deficit in AD and its impact on pathophysiology remains to be fully clarified. Age-related cellular senescence and cerebrovascular diseases are major risk factors for AD, and the accumulation of pathologic protein aggregates in AD brains could activate innate immune responses, leads to chronic inflammation within basal forebrain (BF); this is detrimental to the vulnerable cholinergic system, which is linked to cholinergic deficits in modulating neurovascular function. To test this hypothesis, the rCBF haemodynamics to exogenous ACh were assessed in the neocortex of middle-aged and old 3xTgAD and WT mice. Additionally, tau and amyloid-ß pathologies, Iba1^+^ microglia phenotypes and the senescence markers (p16^Ink4ɑ^ and p21^cip1^) were examined within BF of 3xTgAD mice. Our study revealed that neocortex CBF haemodynamics in response to exogenous ACh were altered in AD mice, suggesting cholinergic deficit. Microglia, the major effectors of innate immunity, interact with tauopathy and increased SASP (senescence-associated secreting phenotype). This, in turn, amplifies local chronic neuroinflammation and senescence, which are associated with cholinergic pathophysiology.

## Materials and methods

### Animals

The 3xTgAD mice were generated by the co-integration of two transgenes encoding the human tauP301L mutant (MAPT, 4R0N, proline mutated to leucine at residue 301) and the Swedish double mutation of APP_Swe_ (APPKM670/671NL). Both were under the control of mouse Thy1.2 promotor and incorporated into embryos of homozygous mutant PS1_M146V_ knock in (PS1-KI) mice.^32^ The 3xTgAD mice used in the present study were crossed onto a congenic C57BL/6 background. The mutant genes were confirmed by genotyping; the characteristic pathology associated with amyloid-β and mutant human tau protein was confirmed by immunohistochemistry and Western blot evaluation, as described previously.^33^ Alzheimer’s murine model (3xTgAD) and age-matched wild type (WT) were investigated in two age groups, middle-aged adult (6–14 months) and old (16–24 months and over). All mice used in this study were males, in the light of concerns that the female hormone (oestrogen) could influence CBF levels and would be expected to change from young to old age. Furthermore, a different oestrogen cycle across individual female mice could potentially increase variance within groups. All in vivo procedures performed on animals were approved by the National Institute on Aging Animal Care and Use Committee (The ACUC protocol: ASP # 488-TGB-2025) and complied with NIH guidelines.

### Pharmacological reagents

Acetylcholine chloride, Cat: A6625, ≥ 99%, (TLC), AG: analysis grade; N^ω^-nitro-L-arginine methyl ester (L-NAME), Cat: N5751, ≥ 97%, (TLC), were from Sigma-Aldrich (St. Louis, MO). BTA-1 (2-(4′-Methylaminophenyl) benzothiazole, Cat: B9934, ≥ 98%, (HPLC), a fluorescent dye of thioflavin-t neutral derivatives rhodamine was obtained from Sigma-Aldrich (St Louis, MO).

### Assessment of regional cerebral blood flow (rCBF) haemodynamics

**Figure 1:**
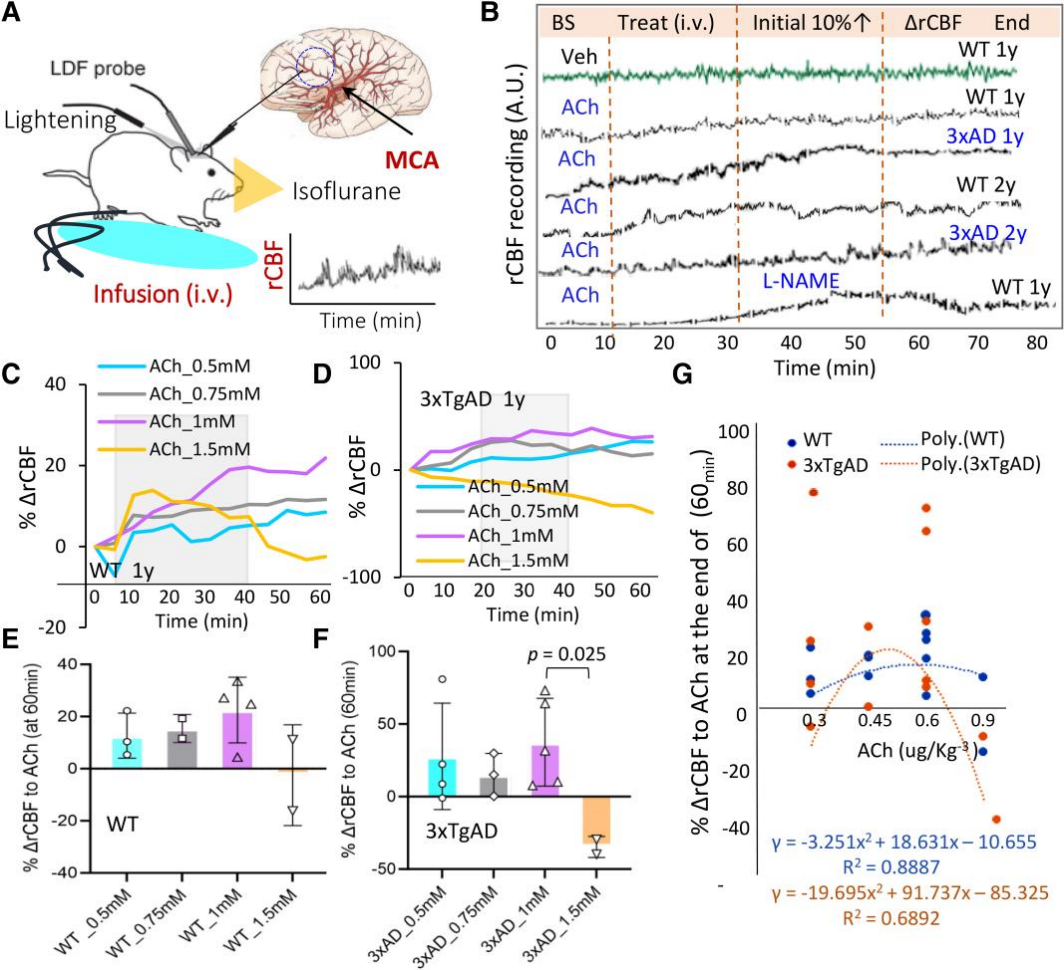
**The rCBF haemodynamics to ACh in WT and AD mice.** (**A**) Mice were maintained under light isoflurane anaesthesia via face mask and the LDF fibre probe was fixed on the thinned cranial window over the brain area perfused by middle cerebral artery (MCA). The haemodynamic traces were monitored and recorded with computer software. (**B)** Representative trace recordings of hemodynamics in response to ACh (0.6 µg/kg^−3^ in saline, i.v.) in 3xTgAD and WT mice from two age groups, and response to L-NAME. (**C, D**) The representative rCBF hemodynamics to doses of ACh (i.v.: 0.5 mM (0.3 µg/kg^−3^), 0.75 mM (0.45 µg/kg^−3^), 1.0 mM (0.6 µg/kg^−3^), and 1.5 mM (0.9 µg/kg^−3^)) were presented as per cent change of rCBF from baseline level (% ΔrCBF) at 5-minute intervals in WT and 3xTgAD mice (∼1y). (**E, F**) The mean % ΔrCBF responses to doses of ACh at the end of recording (∼60 min) in WT and 3xTgAD mice (∼1y). For WT mice, one-way ANOVA analysis on 4 does groups: *F* = 1.91, *P* = 0.217; For 3xTgAD mice, one-way ANOVA analysis on 4 does groups: F = 0.95, *P* = 0.45. The % ΔrCBF response in AD mice was lower at higher dose of ACh (0.9 µg/kg^−3^). *P = 0.025, unpaired two-tailed *t*-test, *t* = 3.164, *df* = 5, *n* = 2–5 mice/group. Data are presented as mean ± SEM. (**G**) The distribution of % ΔrCBF to different doses of ACh from individual mice in WT or 3xTgAD groups at the end of recording (∼60 min). For any questions regarding this document, or other questions about publishing with BioRender refer to our BioRender Publication Guide, or contact BioRender Support at support@biorender.com.

The cortical cerebral blood flow (rCBF) response to various drug treatments was assessed using LDF (PeriFlux System 5000, Perimed, Stockholm, Sweden), as described previously.^30,34^ Mice were lightly anaesthetized with isoflurane in oxygen (∼2% for induction in an anaesthesia chamber, and 1–1.5% for maintenance) during the experimental procedures using a vapour mask. These regimens were consistent throughout the experiments to avoid unequal impact of anaesthesia on rCBF haemodynamics across groups of mice. Animal core body temperature was maintained at 37°C using a thermo-controlled surgical heating plate throughout the experiments and recovery from anaesthesia. The mouse head was immobilized in a stereotaxic frame; an incision and a closed cranial window with thinned skull were created over the cortex posterior and left to Bregma, above the brain region supplied by the left middle cerebral artery (Fig. 1A). This included frontal and lateral parietal cortex, and superior temporal regions. A flexible 0.5 mm diameter fibre optic LDF probe (Perimed; Probe 418, Stockholm, Sweden) was fixed on the thinned cranial window. Drugs were infused into the femoral vein in a volume (100–200 μl) adjusted to body weight at the desired concentration, and then circulated via leptomeningeal/pial arteries, intracerebral arterioles, and capillaries under the cranial window following systemic administration. A catheter line was maintained when repeated drug administrations were needed. The resting rCBF was recorded for at least 5 minutes prior to drug administration to ensure a stable baseline. A dynamic rCBF trace was, thereafter, acquired and recorded for up to 1 hour or longer at 5-minute intervals. After assessment of rCBF response to treatment, the skin incision was closed; buprenorphine was administered (1.0 mg/kg, subcutaneously) to relieve post-operation pain. Data on rCBF responses were analysed by PeriFlur software on haemodynamic values (Perimed) and expressed as per cent change in rCBF related to the baseline level (% ΔCBF).

According to Poiseuille’s formula: CBF = (MAP—ICP) · *πr*^4^/8*η* · *L* (*r* = diameter or resistance or artery; MAP = mean artery pressure; ICP = intra-cranial pressure; *η* = velocity). When MAP, ICP, and η are consistent (no significant change), the ‘r’ diameter or resistance of the artery is the major factor affecting CBF.^30^ Besides, alterations in vessel morphology and existing conditions of brain atrophy could affect CBF.

### Cerebrovascular labelling

Rhodamine B dextran (MW 200,000, Sigma-Aldrich, St. Louis, MO) was prepared in buffered saline (1 mg/ml) and infused into mice intravenously (i.v.). Mice were euthanized, and coronal brain sections were collected by cryostat, and cerebral vascular fluorescence labelling was observed by confocal microscopy (LSM 710, Zeiss). BTA-1 (2-(4′-Methylaminophenyl) benzothiazole (Sigma-Aldrich, St. Louis, MO), an uncharged derivative of thioflavin-t neutral derivatives, was prepared in saline and infused into mice i.v. (10 mg/kg). Mice were euthanized within 2 hours, and brains were removed; fresh frozen serial brain coronal sections were collected and scanned by confocal microscopy (LSM 710, Zeiss). The density of fluorescent labelled vessels (green) within the cortex region was evaluated on multiple brain sections of WT and 3xTgAD mice in each group.

### Immunohistochemistry and confocal imaging

Mice were anaesthetized with isoflurane and first perfused intracardially with ice-cold saline and then with 4% paraformaldehyde in phosphate-buffered saline (PBS), followed by euthanasia. The mouse brains were removed immediately, post-fixed in 4% PFA in PBS overnight, and cryoprotected by soaking in a solution of 30% sucrose in PBS at 4°C. After the brain had equilibrated in the sucrose and sunk to the bottom of the tube, they were stored at −80°C. For immuno-histological assessment, serial sagittal or coronal brain sections were sliced at 25 µm thickness and stored until use. Endogenous peroxidases were quenched by incubation of sections in 0.3% hydrogen peroxide solution. Sections were blocked with 5% normal goat serum or albumin in TBS buffer with 0.1% Triton X-100 (TBST) for 1 hour at room temperature before incubation with the primary antibodies overnight at 4°C. Antigen retriever was applied when needed. Primary antibodies used in these studies included: anti-choline acetyltransferase (ChAT, 1:200, Millipore-Sigma, CA, USA); antibodies recognizing phosphorylated tau (Thr181, 1:200) and human Tau (HT7, T13, 1:500) from Thermo Fisher Scientific; antibodies recognizing β-amyloid 1–16 (6E10, Covance, Dedham, MA); antibodies recognizing microglial cells (Iba-1, 1:400, rabbit anti-human ionized calcium-binding adaptor molecule, Wako, Osaka Japan) or astrocytes (GFAP, Chemicon); Antibodies recognizing p16^Ink4ɑ^ and p21^cip1^ (abcan 1:400) and TNF-α (Abbiotec 1:500). Sections were washed with TBST and incubated with Alexa Fluor-conjugated secondary antibodies (Invitrogen, CA, USA) at room temperature, washed and mounted with VECTASHIELD® Antifade Mounting Medium with DAPI (Vectorlabs, USA). Images were acquired by a confocal microscope (Zeiss LSM710, Germany) using ZEN software, and collected with either a 20 × or 40 × magnification objective lens. NIH Image J 1.52q was used for quantification, including an area with a positive signal in defined brain region. Confocal tiling scanning was applied for labelled cell evaluation within defined brain regions (such as at cholinergic nuclei in BF). The boundaries of brain regions investigated in this study are defined according to the mouse brain atlas^35^ and indicated (Supplementary Fig. 1). Controls consisted of omission of primary antibodies and evaluation by blinded observers.

### Microglia phenotyping at basal forebrain

Morphology phenotyping of Iba1labelled microglial cells was performed on serial brain sagittal sections including the BF region in 3xTgAD and WT mice (*n* = 4–7 mice/group). Images from BF were collected by 20 × or 40 × (oil magnification) objective lenses from 2 to 4 sections/mouse for the average percentage of each phenotype of Iba1^+^ microglia in each group. Heterogenous morphologies of microglia were phenotyped into 4 categories: (i) Ramified *(resting, surveillance)*: small somas, long and thin branched processes; (ii) Hypertrophic *(activated, intermediate or bush*): enlarged soma with highly branched or rod processes; (iii) Dystrophic *(senescent*): de-ramified, retracted processes, shortened branches with enlarged or twisted soma; branches could be ‘beaded’ or with spherical swelling ends and fragmentation. (iv) Amoeboid *(immature, or active phagocytosing)*: round cell bodies, which appear to be retracted or devoid of processes, representing reactive phagocytosis microglia or during development. The average percentages of each microglia phenotype within total microglia per image collected within BF from each group of 3xTgAD or WT mice were evaluated for statistical analyses.

The subtleties of Iba1^+^ microglia morphology were further quantitatively characterized by Fractal analysis,^36^ with NIH Image J 1.52q and MotiQ software with 40 × magnification collected images as described previously.^37^ Individual microglial cells were randomly chosen within BF to identify differences in microglia morphology between 3xTgAD and WT mice in each group (5–6 cells/image; 2–3 images/per mouse, *n* = 4 animals/each group were evaluated). Multiple morphological parameters were analysed for individual microglia including ramification index, spanned area, number of branches, junctions, endpoint, and total tree length of microglia in BF of each animal from WT or 3xTgAD groups.

### Immunoblot

Mouse brain cortex tissues were prepared for Western blot analysis as described previously.^33^ Briefly, brain tissues were homogenized and solubilized in sample buffer (Invitrogen) containing cOmplete^TM^ protease inhibitor and phosphatase inhibitor cocktails (Roche Diagnostics, Indianapolis, USA). Protein concentrations were determined by use of a BCA assay (Pierce, Waltham, MA, USA); protein samples were separated on precast 4–12% NuPAGE gradient gels (Invitrogen, USA) by electrophoresis, then transferred to a nitrocellulose membrane and blocked in 5% nonfat milk in Tris-buffered saline with 0.05% Tween 20. The blots were incubated with the primary antibodies overnight at 4°C. The primary antibodies used were anti-ChAT antibody (1:500; Millipore-Sigma), anti-synaptophysin antibody (1:1000. Abcam), and β-actin (1:2000; Sigma-Aldrich). After washing, the membranes were incubated with an HRP-conjugated secondary antibody (Vector Laboratories, Burlingame, CA, USA) for 1 hour at room temperature. Blots were developed using an ECL chemiluminescence reagent (Pierce, Rockford, Illinois, USA). The integrated density of protein bands was quantified using Image J software (NIH) and normalized to the density of the β-actin band on the same blot. (Supplementary Fig. 5)

### Statistical analysis

The GraphPad Prizm 10.0.3 (San Diego, CA, USA) and ‘R’ software (version 4.2.2; 2022–10–3, ucrt) were used for statistical analysis and graphs. Data on rCBF haemodynamics were recorded by PeriFlur software (Perimed), represented as percentage changes of rCBF (% ΔCBF) related to baseline level within time intervals and plotted as mean ± SEM (standard error of the mean); means of % ΔCBF with 95% confidence interval (CI) were presented on Table 1. Probability *P*-value ≤ 0.05 was considered statistically significant. For image analysis for intergroup differences between WT and 3xTgAD mice, each including the two age groups, analysis of variance (ANOVA) was performed. Equality of data variance was determined by *F*-test. Multiple comparisons between two groups were analysed via unpaired *t*-tests with one- and two-tailed accordingly (GraphPad Prizm10.0.3). NIH Image J 1.52q (particle analysis with defined threshold) was used for quantification on percentages of area with pathology Tau or amyloid β deposition at defined regions of 3xTgAD mice. Fuji Image J/MotiQ software was used for comparisons on subtleties of Iba1^+^ microglia morphology characterized by Fractal analysis on multiple collected confocal images within basal forebrain of age-matched 3xTgAD and WT mice (3 section/mouse; *n* = 4 mice/group). For colocalization evaluation with double/triple immunohistochemistry staining on brain sections, Pearson’s correlation coefficient (*R_p_*) evaluation was applied using Zeiss confocal microscope (LSM710) ZEN software on ROI (region of interesting) from multiple images for statistical analysis (ZEISS: Acquiring and Analysing Data for Colocalization Experiments in AIM or ZEN Software). Probability *P*-value < 0.05 was considered statistically significant. Accordingly, the value of Pearson correlation coefficient (r) was interpreted as *r* > 0.5 as strong positive; *r* between 0.3 and 0.5 as moderate positive; r between 0 and 0.3 as weak positive; 0 as none, while *r* below 0 as negative. The Bonferroni correction was used for serial measurements if needed.

**Table 1:** The parameter of rCBF haemodynamics to ACh in 3xTgAD and WT mice

Parameters	Middle-aged		Old	
	WT (*n* = 6)	3xTgAD (*n* =6 )	WT (*n* = 6)	3xTgAD (*n* = 5)
Initial *T*_(min)_ of rCBF↑^a^	24.167 (15.25–33.08)	11.667 (6.82–16.51)*	26.667 (12.88–40.45)	31 (7.73–54.24)
Peak of % ΔrCBF^b^	22.702 (15.5–29.91)	48.35 (27.03–69.67)*	23.252 (7.03–39.47)	21.436 (2.52–40.35)^†^
End of % ΔrCBF↑^c^	20.14 (11.59–28.69)	31.24 (6.44–56.04)	19.127 (3.57–34.68)	11.668 (−6.3–32.63)

Data are mean (95% CI), analysed within LDF recording periods (60 min) after ACh administration (i.v.).

^a^ *T*_min_ is the time (minute) to initiate a 10% rCBF increase from baseline level following drug (ACh) administration.

Note in old mice, a few mice (1 in WT, 2 in 3xTgAD) were not able to reach a 10% rCBF increase within 60 min.

^b^The peak of % ΔrCBF from baseline level after ACh administration (i.v.) within the recording period (60 minute).

^c^The % ΔrCBF at the end of the recording period (60 minute) in response to ACh administration (i.v.).

^*^ *P* ≤ 0.05 between age-matched WT and 3xTgAD mic; 2-sample *t*-test, 2-tailed.

^†^ *P* ≤0.05 between middle-aged AD and old AD mice; 2-sample *t*-test, 1-tailed.

## Results

### Cortical rCBF haemodynamic response to ACh in WT and AD mice

The dose–response relationships of cortical rCBF response to ACh administration (i.v.) in WT and AD mice were evaluated. Mice were maintained under light isoflurane throughout the procedure and rCBF was reported by an LDF fibre through a thinned cranial window over the cerebral region supplied by the middle cerebral artery (MCA) (Fig. 1A). Representative traces on rCBF haemodynamics in response to ACh (0.6 µg/kg^−3^ in saline, i.v.) were recorded in 3xTgAD and WT mice from two age groups over a duration of 60 minutes in 5-minute intervals (Fig. 1B). To determine whether nitric oxide (NO) is involved in the signalling of the rCBF response to ACh, the nitric oxide synthase (NOS) inhibitor N^ω^-nitro-L-arginine (L-NAME) was given before ACh administration (i.v.), which partially attenuated rCBF elevation (Fig. 1B). Four concentrations of ACh: 0.5 mM (0.3 µg/kg^−3^), 0.75 mM (0.45 µg/kg^−3^), 1.0 mM (0.6 µg/kg^−3^), and 1.5 mM (0.9 µg/kg^−3^) were time-dependently evaluated in middle-aged mice, with representative rCBF responses shown in WT (Fig. 1C) and 3xTgAD (Fig. 1D) mice, respectively. The mean % ΔrCBF at the end of recording (∼ 60 minutes) to different doses of ACh in WT (Fig. 1E) and 3xTgAD (Fig. 1F) mice demonstrated dose-dependent elevations in % ΔrCBF in WT and 3xTgAD mice, which declines at a highest ACh dose (1.5 mM (0.9 µg/kg^−3^)) as plotted (Fig. 1G). Based on this study, an ACh dose of 0.6 µg/kg^−3^ was used in subsequent studies.

### The rCBF haemodynamics to ACh altered early in neocortex of AD mice which linked to cholinergic deficits

**Figure 2:**
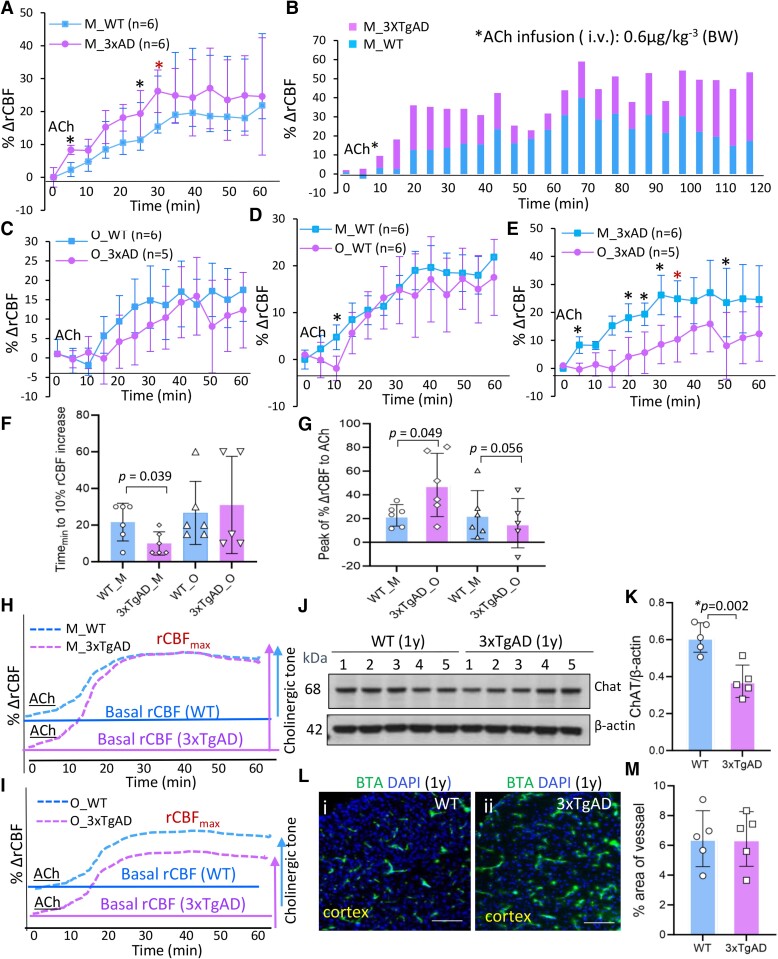
**Age-related rCBF haemodynamics to ACh in WT and AD mice.** (**A**) The mean % ΔrCBF to ACh (0.6 µg/kg^−3^, i.v.) was higher in middle-aged AD (magenta) versus in age-matched WT (cyan) mice at several time points *P < 0.05, *n* = 5–6 mice/group, two-tailed *t*-test with equal variance (red); or 1-tailed *t*-test (black). (**B**) Representative % ΔrCBF to ACh lasted for 2 hours in middle-aged WT (M-WT) and AD mice (M-3xAD). (**C**) The % ΔrCBF to ACh in old 3xTgAD (O_3xAD) versus old WT (O_WT) mice. (**D**) The % ΔrCBF to ACh between middle-aged (cyan) and old WT (magenta) mice were not significantly different. (**E**) The % ΔrCBF to ACh was reduced at multiple time points in old (magenta) compared to middle-aged (cyan) 3xTgAD mice. *P < 0.05, *n* = 5–6 mice/group, 2-tailed *t*-test (red); 1-tailed *t*-test (black). (**F**) Time to initiate 10% ΔrCBF increase to ACh in four groups. One-way ANOVA: *F* = 1.761, *P* = 0.188. It was faster in middle-aged 3xTgAD versus in WT mice. *P = 0.039; *t* = 2.36, *df* =10, *n* = 6 mice/group; 2-tailed *t*-test with equal variance. (**G**) The mean peak of % ΔrCBF to ACh in four groups: 1-way ANOVA: *F* = 2.86, *P* = 0.064. The peak of % ΔrCBF was higher in middle-aged 3xTgAD versus in age-matched WT mice. *P  *=* 0.049, *n* = 6 mice/group, unpaired 2-tailed *t*-test, *t* = 2.234, *df* = 10. The peak of % ΔrCBF was reduced in old versus in middle-aged AD mice but not significant, *P*  *=* 0.056, *t* = 2.198, *df* = 9. (**H**) Hypothetic illustration on % ΔrCBF to ACh in 3xTgAD and WT mice from two age groups. The basal rCBF level was lower in middle-aged AD versus in WT mice. The % ΔrCBF to ACh was initiated from a lower baseline in AD mice but was able to reach the maximal rCBF level as in WT mice, given the preserved cerebrovascular reactivity to ACh in AD mice. (**I**) The % ΔrCBF to ACh was reduced in old AD than in middle-aged AD mice, and rCBF level was not able to reach the maximal rCBF level, even the % ΔrCBF was not different within old AD to old WT mice. (**J–K**) The protein levels of ChAT in neocortex from 3xTgAD and WT mice (1y) were assessed by western blot and normalized to β-actin in the same blot. Ratio of integrated protein band density of ChAT to β-actin (Image J), *P*  *=* 0.002, unpaired two-tailed *t*-test; *t* = 4.47, *df* = 8, *n* = 5 mice/group. (**L–M**) Representative images of BTA labelled vascular density in cortex regions from multiple images of age-matched WT (Li) and 3xTgAD (Lii) mice. unpaired two-tailed *t*-test, *P* = 0.985, *t* = 0.0184, *df* = 8; 2–3 mice/group. Scale bar, 100 µm.

The cerebrovascular responses of rCBF to ACh and the impact of age were evaluated in two age groups of 3xTgAD and WT mice. The elevation of % ΔrCBF to ACh (0.6 µg/kg^−3^, i.v.) was initiated earlier with higher amplitude at several time points (10–20% higher) in middle-aged 3xTgAD versus age-matched WT mice (*P ≤ 0.05, *n* = 6 mice/group, Fig. 2A). The elevated rCBF levels in response to ACh administration lasted 2 hour’s duration in middle-aged 3xTgAD and WT mice (Fig. 2B). In contrast, in old 3xTgAD mice, the mean % ΔrCBF to ACh tended to be lower than in age-matched old WT mice, although it did not reach statistical significance (Fig. 2C), but it was significantly lower than the that in middle-aged 3xTgAD mice (Fig. 2E, *P ≤ 0.05, *n* = 5–6 mice/group). There was no significant age difference in mean % ΔrCBF between middle-aged and old WT mice (Fig. 2D). The onset of the rCBF response to ACh (time to 10% increase of rCBF from baseline level) was faster in 3xTgAD from the middle-aged group (Fig. 2F, *P < 0.05). The peak of % ΔrCBF to ACh was higher in middle-aged AD mice versus middle-aged WT mice but reduced in old 3xTgAD mice (Fig. 2G). Notably, the concentrations of isoflurane used in the assessment, (which has an impact on rCBF), were not significantly different between age-matched WT and 3xTgAD mice.

The early enhanced % ΔrCBF response and faster initiation to ACh in younger 3xTgAD was potentially due to a deficiency of endogenous ACh because of the denervation of cholinergic input in neocortex of 3xTgAD mice. As hypothetically illustrated (Fig. 2H–I), the basal level of rCBF (modulated by cholinergic tone) is lower in middle-aged 3xTgAD mice (magenta) versus in age-matched WT mice (cyan), while the cerebrovascular reactivity to external ACh administered i.v. was preserved and could respond to the maximal rCBF levels (Fig. 2H), primarily via signalling on ACh receptors in the cerebral vasculature.^13-15^ In old 3xTgAD mice (Fig. 2I), even though the % ΔrCBF to ACh was not significantly lower than in old WT mice (magenta); it was unable to reach the maximal rCBF perfusion levels because of the lower basal level of rCBF and cholinergic tone. The differences in the rCBF haemodynamic responses to ACh (including 95% CI) from the two age groups of WT and AD mice are listed in Table 1.

To investigate the mechanism of cholinergic deficits in 3xTgAD mice, ChAT, the choline acetyltransferase enzyme involved in ACh synthesis, was evaluated in neocortex samples from WT and AD mice. ACh is synthesized in the soma of cholinergic neurons, transported to the synaptic terminal and released at the synaptic cleft; it then binds to ACh receptors in vascular cells and triggers downstream signalling. ACh is hydrolysed by acetylcholinesterase (AChE) into acetate and choline, recycled into the presynaptic nerve terminal by high-affinity choline transporters. Immunoblots revealed ChAT levels were reduced early in 3xTgAD (1y) in cortex tissues compared to WT mice (Fig. 2J–K, *P* < 0.01, 2-tailed *t*-test, *n* = 5 mice/group). With respect to the possible changes of cortical vascular densities on the rCBF response, cerebrovascular density was evaluated in AD and WT mice via BTA labelling (i.v.). BTA is an uncharged derivative of thioflavin-T with brain entry and clearance to provide high-affinity binding to Aβ fibrils. The cortical density of fluorescence-labelled vessels was not significantly different in 3xTgAD versus WT mice ∼1y (Fig. 2L–M). Increased cerebral vascular density with vessel abnormality and angiogenesis-related gene expression have been reported previously in P301L transgenic mice and human Alzheimer’s disease.^38^

### Tauopathy deposits early within selected ChAT^+^ cell clusters of cholinergic nuclei at basal forebrain of AD mice

**Figure 3:**
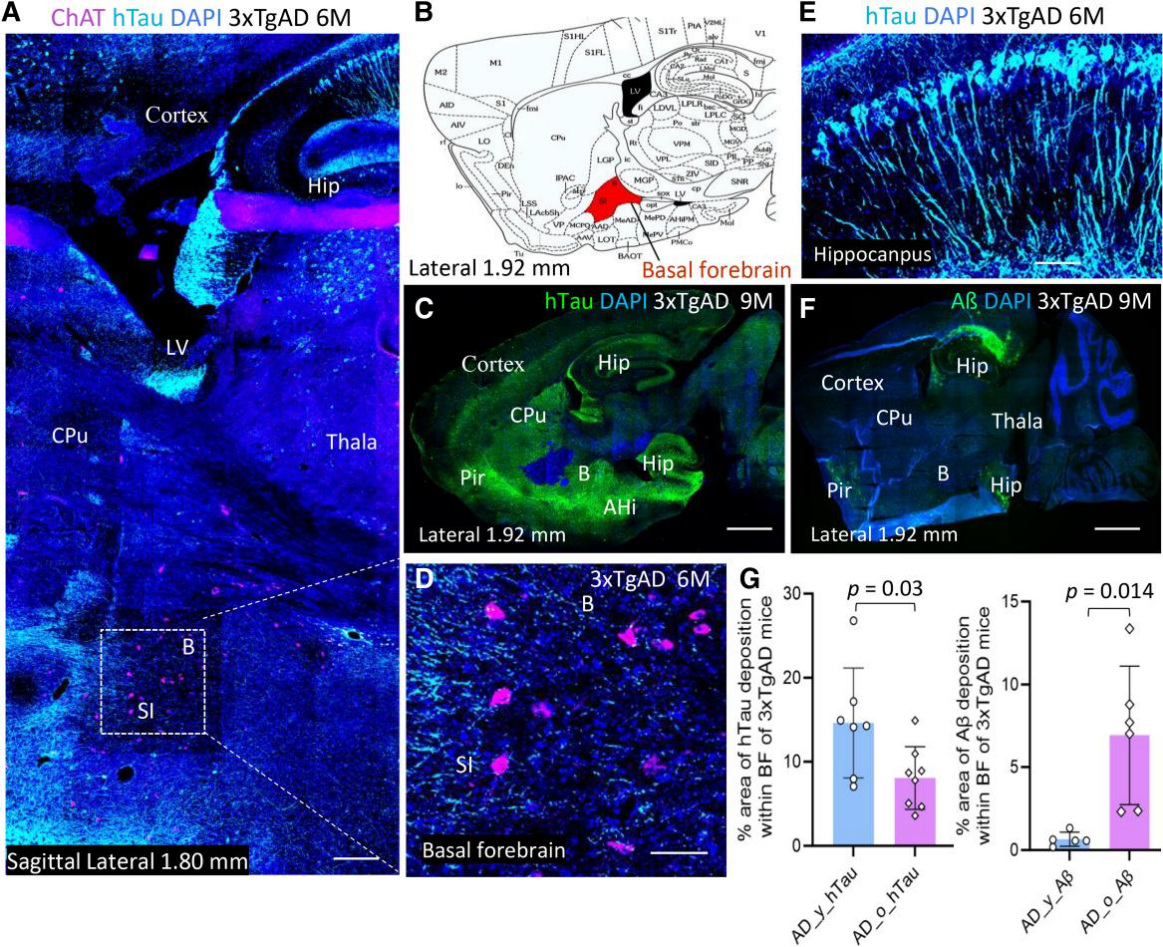
**Early deposition of tauopathy localized within selected ChAT^+^ cell clusters in BF of 3xTgAD mice.** (**A**) Representative sagittal brain image of a 3xTgAD mouse (6 M), showing ChAT^+^ cells (magenta) and mutant hTau (cyan) within the BF including nBM (B) and SI. (**B**) The location of nBM and SI in BF is highlighted (orange) in a sagittal map of mouse brain atlas.^35^ (**C**) Representative tiling image showing mutant hTau (green) deposition on a full sagittal section of a young 3xTgAD mouse (9 M). (**D)** Immunofluorescence labelled hTau (cyan) and ChAT (magenta) within the BF of 3xTgAD mouse (6 M) in higher magnification. **(E)** hTau (cyan) expression at hippocampus of AD mouse. (**F**) Regional tiling image of amyloid-β deposition (green) on a sagittal section of a 3xTgAD mouse (9 M), mainly at hippocampus and neocortex but not evident in BF. (**G**) The percentages of area (%) with hTau or amyloid β deposition within BF of 3xTgAD mice were evaluated in two age groups. The area (%) with hTau deposition was higher in young versus in old AD mice, *P* = 0.031, unpaired two-tailed *t*-test with equal variance, *n* = 4–6 mice/group; *t* = 2.428, *df* = 13; *F* = 3.96 (6,7), *P* = 0.165. The % area of amyloid β deposition was higher in old versus in young AD mice. *P* = 0.014, unpaired 2-tailed *t*-test with Welch’s correction for unequal variance (GraphPad Prism10.0.3), *n* = 3–5 mice/group; *t* = 3.648, *df* = 5.120; *F* = 99.52, *P* < 0.01. Abbreviations: BF: Basal forebrain; SI: Substantia innominate; nBM (B); nucleus Basalis of Meynert; CPu: Caudate putamen. Scale bar, 400μm in panel **A**; 750μm in panel **C** and **F**; 50μm in panel **D** and 100μm in panel **E**.

The cortical cholinergic innervation is derived from cholinergic projection neurons within selected nuclei within the BF, mainly from the nucleus basalis of Meynert (nBM).^31^ Since a deficit of endogenous ACh could be relate to the cholinergic denervation or loss from the cholinergic system within the BF, fluorescence immunohistochemistry for ChAT, a cholinergic cell marker; and tauopathy (human mutant tau P301L and phosphorylated tau at Thr181), which is associated with neurodegeneration in AD patients and rodent models,^39,40^ were examined in BF of 3xTgAD and WT mice. ChAT^+^ cells located within the BF near and below the CPu of striatum in representative images on sagittal sections of 3xTgAD mice (Fig. 3A and D), and pathological tau protein appeared as early as 4–6 months of age within the BF of 3xTgAD mice (Fig. 3C; Supplementary Fig. 2) prior to the appearance of extracellular Aβ plaques in the BF of AD mice (Fig. 3F; Supplementary Fig. 3). Deposits of tauopathy distributed in entorhinal cortex, hippocampus, neocortex and amygdala in young 3xTgAD mice (Fig. 3A, C and E),^33^ recapitulate tau pathology manifested in human AD as Braaks stages I–III.^41^ Notably, amyloid-β pathology (extracellular diffuse or neuritic plaques) appeared early within the hippocampus (Fig. 3F) but was not evident in the BF until a later stage (Supplementary Fig. 3). The area (%) of pathology tau and amyloid β deposition within BF was evaluated in 3xTgAD from two age groups (Fig. 3G). The area (%) with tauopathy was higher in young versus in old AD mice (*P* = 0.03, unpaired 2-tailed *t*-test with equal variance, *t* = 2.428, *df* = 13; *n* = 3–5 mice/group), with loss of hTau positive fibrils in old AD mice (Supplementary Fig. 2). In contrast, the area (%) with amyloid β plaques was significantly increased in BF of old AD mice compared to young AD mice (Fig. 3G), *P*  *=* 0.014, unpaired two-tailed *t*-test with Welch’s correction of unequal variances (*t* = 3.648, *df* = 5.12, *n* = 3–5 mice/group).

**Figure 4:**
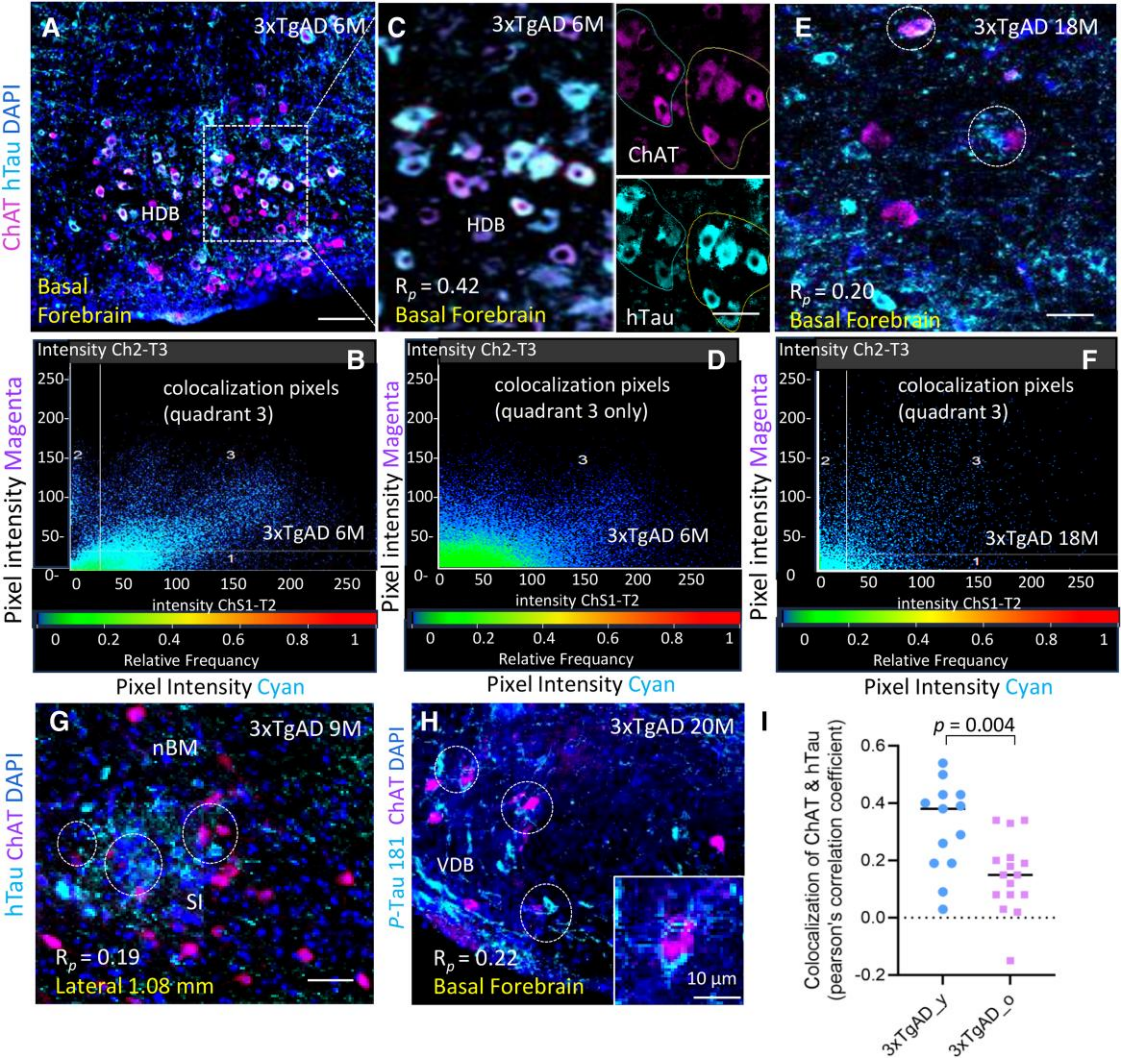
**Coexistence of tauopathy (hTau and pTau 181) in selected ChAT^+^ cell clusters within BF of 3xTgAD mice**. (**A**) Representative image showing mutant hTau (cyan) deposition in selected ChAT^+^ cell cluster (magenta) at HDB on a sagittal section of a young 3xTgAD mouse (6 M). (**B**) Scatterplot of pixel intensities of cyan (quadrant 1), magenta (quadrant 2) and colocalization pixels (quadrant 3). (**C–D**) Higher magnification image showing colocalization of hTau and ChAT^+^ at HDB and its colocalization pixels at quadrant 3 only on scatterplot. Pearson’s correlation coefficient (R*_p_*) = 0.42 (ROI in circle). (**E–F**) Representative image on hTau (cyan) and ChAT^+^ (magenta) expressing at BF from an old 3xTgAD mouse(18 M) and its scatterplot; R*_p_* = 0.20 on ROI. (**G**) Representative image on hTau deposition within ChAT^+^ cell clusters at nBM and SI from a young 3xTgAD mice (9 M), R*_p_* = 0.19. (**H)** Representative image on phosphor-tau Thr181 (cyan) deposition within ChAT^+^ cell clusters at VDB in an old 3xTgAD mouse (20 M), R*_p_* = 0.22. (**I**) Average R*_p_* on colocalization of hTau and ChAT^+^ cells at ROI within BF from multiple brain sections of young and old 3xTgAD mice (each dot represents one ROI). The average R*_p_* was higher in young versus in old AD mice. *P* = 0.004, unpaired 2-tailed *t*-test with equal variance, *t* = 3.205. *df* = 27; *F* = 1.476, *P* = 0.471. *n* = 3–5 mice/group. Abbreviations: R*_p_*: Pearson’s correlation coefficient. ROI: region of interesting. nBM: nucleus Basalis of Meynert; HDB: nucleus of horizontal limb diagonal band of Broca; VDB: nucleus of vert limb diagonal band. Scale bar, 200μm in panel **A**; 100μm in panel **C**, **E**, **G** and **H**.

The coexistence of tauopathy in BF within selected ChAT^+^ cell clusters of cholinergic nuclei was observed occasionally in BF of 3xTgAD mice, including the SI (substantia innominata), nBM (nucleus Basalis of Meynert), and HDB (nucleus of horizontal limb diagonal band of Broca) on regions of interest (ROI, indicated by circle) at sagittal brain sections (Fig. 4A–G). The phospho-tau (pTau181) deposition and spreading fibrils in neuronal cells were also observed in the vicinity of ChAT^+^ cells within the BF of AD mice (Fig. 4H). However, the colocalizations of tauopathy with ChAT^+^ cells were not particularly abundant. The colocalization of hTau and ChAT at BF was evaluated in young and old AD mice by Pearson’s correlation coefficient (*R_p_*) on ROI, and the average *R_p_* assessed on multiple ROI within BF was higher in young versus in old 3xTgAD mice (Fig. 4I): *P* = 0.004, unpaired 2-tailed *t*-test with equal variance, *t* = 3.205, *df* = 27, *n* = 3–5 mice/group.

### Iba1^+^ microglia were interacting with tauopathy, exhibiting phenotypic changes in the BF of AD mice

**Figure 5:**
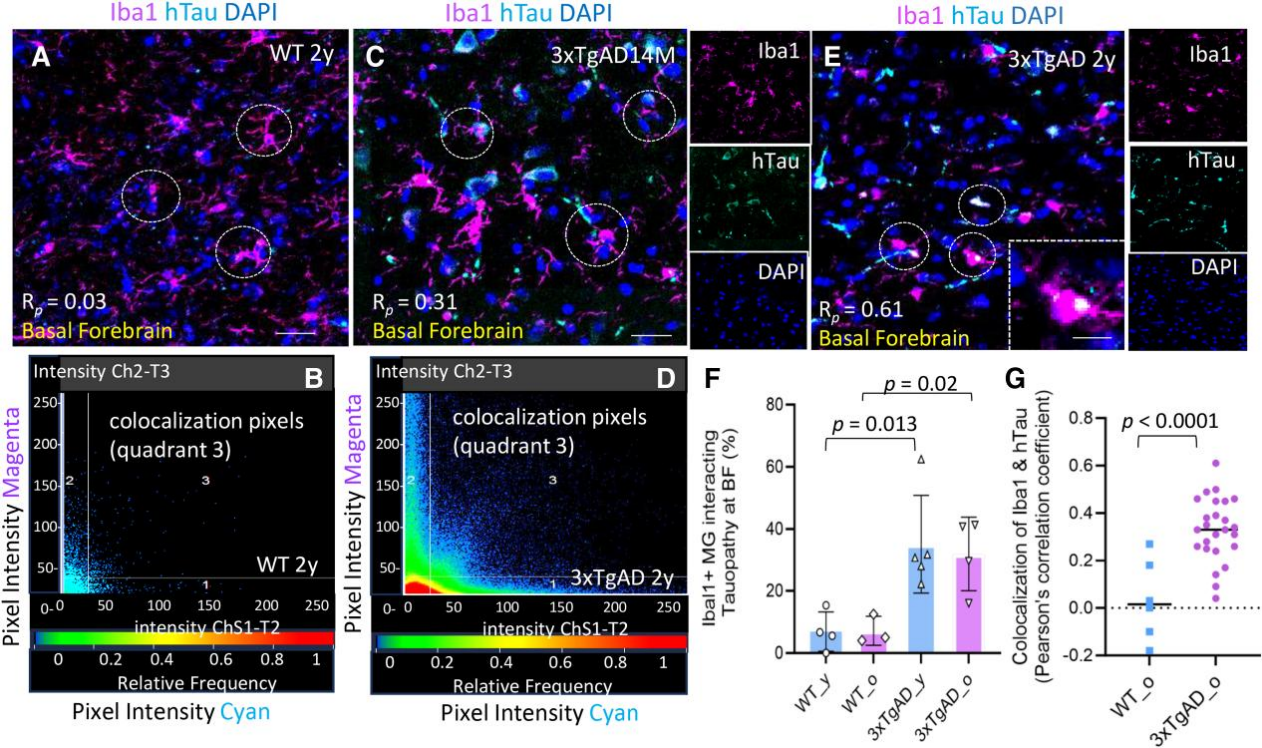
**Iba1^+^ microglia interacting tauopathy with phenotypic changes in BF of 3xTgAD mice.** (**A–B**) Representative images show Iba1^+^ microglia (magenta) at BF of an old WT mouse (2y) and scatterplot of colocalization pixels of Iba1(magenta) and hTau (cyan), which was almost undetectable in WT mice, R*_p_* = 0.03. (**C–D**) Representative images reveal Iba1^+^ microglia (magenta) were interacting hTau (cyan) within BF of a 3xTgAD mouse (14 M) and scatterplot on colocalization pixels at ROI. R*_p_* = 0.31. (**E**) Internalization, engulfing or phagocytosing hTau by Iba1^+^ microglia at BF of an old 3xTgAD mouse (2y). R*_p_* = 0.61. Microglia also showed heterogeneous morphology. (**F**) The average % of microglia associated with tauopathy (including phagocytosis) at BF was higher in 3xTgAD mice versus Age-matched WT mice. One-way ANOVA, *F* = 7.105, *P* = 0.005. Multiple comparison using unpaired 2-tailed *t*-test with equal variance between young WT and 3xTgAD mice, *P* = 0.013, *t* = 3.32, *df* =7; between old WT and 3xTgAD mice, *P* = 0.02, *t* = 3.5, df =5; *n* = 3–5 mice/group. (**G**) The average R*_p_* on colocalization of Iba1 and tauopathy at ROIs within BF from multiple brain sections of old 3xTgAD and WT mice, *P* < 0.0001, unpaired 2-tailed *t*-test with equal variance; *t* = 4.634, *df* =29. *F* = 1.567, *P* = 0.414. *n* = 3–5 mice/group. Scale bar, 100μm in panels 5A-a, c, e.

A prominent pathologic feature noted here in 3xTgAD mice was the presence of reactive Iba1^+^ microglia interacting with pathological tau protein within the BF. This was manifested as an interaction and internalization of tauopathy (hTau or pTau181), engulfing/or phagocytosis in 3xTgAD mice (Fig. 5C and E). About 30% of Iba1^+^ microglia were interacting with tauopathy in young or old AD mice, which was significantly higher than in WT mice, *P* < 0.05 (Fig. 5F). The expression of hTau and pTau181 was not evident in BF of WT mice (Fig. 5A). The average *R_p_* on colocalization of hTau in Iba1^+^ microglia cells were evaluated on ROI from multiple brain sections, which was significantly higher in old AD versus in WT mice (Fig. 5A and G), *P* < 0.01, unpaired 2-tailed *t*-test with equal variance, *n* = 3–5 mice/group. Microglia in AD mice also demonstrated heterogeneity in microglia morphology, especially in those located in the immediate vicinity of tauopathy (Fig. 5C and E).

**Figure 6:**
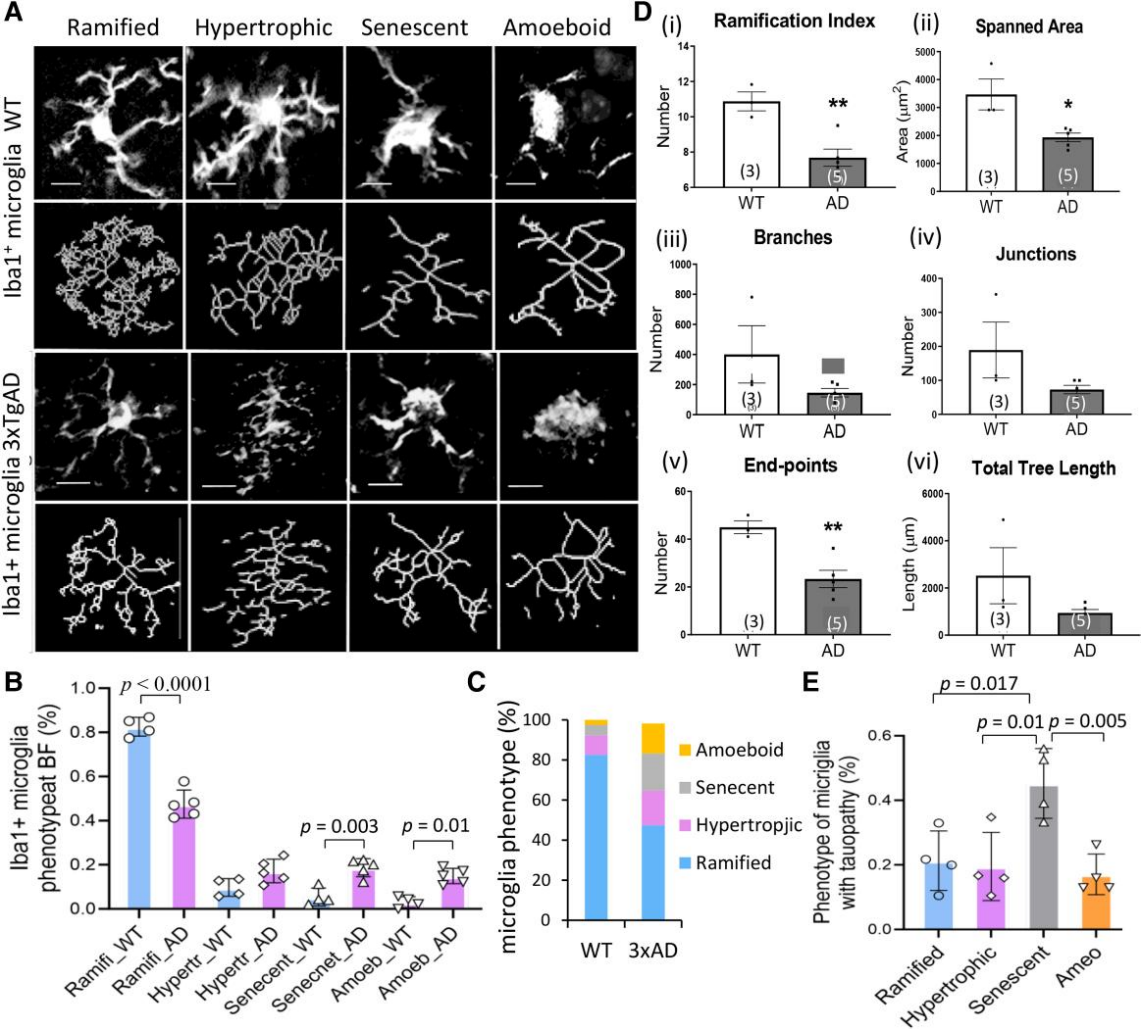
**Iba1^+^ microglia in the BF of 3xTgAD mice exhibited a decrease in ramified phenotype and an increase in dystrophic/senescence phenotype.** (**A**) Heterogeneous morphological phenotypes were evaluated in BF of WT and 3xTgAD mice (16–24 months), including ramified/surveillance, hypertrophic/activated, dystrophic/senescent and amoeboid/phagocytic. (**B–C**) Analysis on microglia phenotypes (%) within BF of 3xTgAD mice. One-way ANOVA analysis, F = 150.3, *P* < 0.0001; Tukey’s multiple comparisons: ramified phenotype (%) was reduced (*P* < 0.001); while senescent (*P* = 0.003) and amoeboid (*P* = 0.01) were increased in 3xTgAD mice compared to WT mice, *df* = 28. (**D**) The morphology of microglia was characterized by Fractal analysis on ramification index (**i**), spanned area (ii), branches (iii), junctions (iv), endpoints (**v**) **a**nd total tree length (vi). Data were evaluated from 3–4 sections/mouse. Two-tailed *t*-test was used between WT and 3xTgAD groups, *P < 0.05; **P < 0.01; *n* = 3–5 mice/group. (**E**) The dystrophic/senescent phenotypes were especially higher in Iba1^+^ microglia located in the immediate vicinity with tauopathy in AD mice (Fig. 5B–E). One-way ANOVA, *F* = 7.766, *P* = 0.0038; Tukey’s multiple comparisons: senescent phenotype (%) was higher than ramified (*P* = 0.0167), hypertrophic (*P* = 0.011) and amoeboid (*P* = 0.005), *df* = 12. Each dot represents mean of each phenotype from multiple sections/each mouse. Scale bar, 10μm in panel 5B-a.

Microglia phenotyping was performed on multiple brain sections from late middle-aged to old 3xTgAD and age-matched WT mice. Four Iba1^+^ microglia phenotypes were evaluated (Fig. 6A), including (1) ramified/resting; (2) hyper-ramified/reactive; (3) dystrophic/senescent; and (4) amoeboid/developing or activated phagocytic, as classified from previous publications.^37,42,43^ The average percentage of each phenotype from 3xTgAD and WT groups revealed a reduced ramified phenotype and an increased hypertrophic, dystrophic/senescent and amoeboid phenotypes within the BF of 3xTgAD versus WT mice (Fig. 6B–C). *P < 0.05, ***P < 0.001; unpaired 2-tailed *t*-test, 3–4 images/each animal, *n* = 4–5 mice/group). Dystrophic/senescent microglia were increased particularly as a fraction of the population for those cells located in proximity with pathologic tau protein (Fig. 6E); characterized by irregular enlarged cytoplasm, swollen or fragmented soma, and reduced ramifications (Figs 5E and 6A).

**Figure 7:**
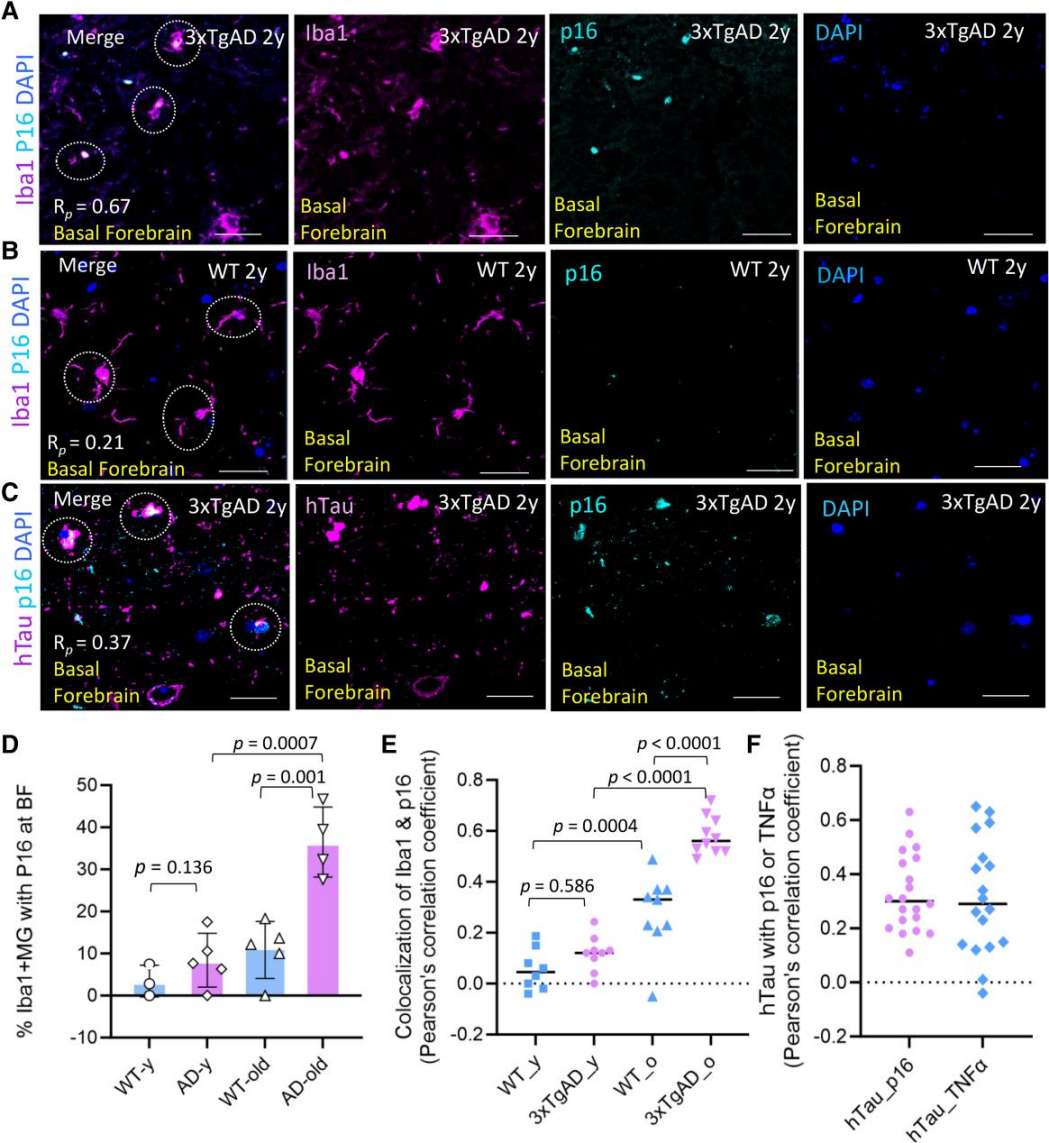
**Increased expression of the senescence marker p16^INK4A^ in Iba1^+^ microglia and hTau harbouring cells with aging in BF of 3xTgAD mice**. (**A–B**) Representative Immunofluorescence images revealed cell senescence marker p16^INK4A^ (cyan) expressed in Iba1^+^ (magenta) microglia within BF of aged 3xTgAD mice (*R_p_* = 0.67), compared to aged WT mice (*R_p_* = 0.21). (**C**) p16^INK4A^ expression (cyan) was also observed in hTau-bearing (magenta) cells within BF of 3xTgAD mice (R*_p_* = 0.37). (**D**) The Iba1^+^ microglia expressing p16^INK4A^ (%) were evaluated within four groups, one-way ANOVA: *F* = 18.99, *P* < 0.0001. It was significantly higher in old versus in younger AD mice, *P* = 0.0007, *t* = 5.755, *df* = 7, and higher in old AD versus old WT mice, *P* = 0.001, *t* = 5.117, *df* = 7; unpaired 2-tailed *t*-test with equal variance, The data points were mean on 3–4 brain sections/each mouse, *n* = 3–5 mice/group. (**E**) The average R*_p_* on colocalization of Iba1 with p16^INK4A^ (ROIs) from multiple sections of WT and 3xTgAD mice in two age groups was compared. One-way ANOVA analysis: *F* = 51.36, *P* < 0.001. The average *R_p_* was significantly higher in old AD versus in young AD mice, and in old AD versus old WT mice, but was not significantly different between young AD versus young WT mice as indicated. Tukey’s multiple comparisons test with adjusted values of *P* < 0.01 was indicated, *df* = 32; *n* = 3–4 mice/group. (**F**) The average *R_p_* on colocalization of p16^INK4A^ or TNF-α expression in hTau-bearing cells at BF of old AD mice. Scale bar, 50μm in panels **A–C**; BF, Basal forebrain.

The ramification index analysis by Image J and MotiQ software further showed the characteristics of Iba1^+^ microglia morphologic features within the BF (Fig. 5B–D), including reductions in (i) ramification, (ii)spanned area, (iii) branches, (iv) junctions, (v) endpoints and (vi) total tree length in mice from 3xTgAD and WT groups (*n* = 4 animals/group). In contrast to AD mice, such dystrophic/senescent microglia were less seldom found in age-matched WT mice (Fig. 6B and Fig. 7B), suggesting the increase of dystrophic/senescent phenotype was disease-associated in the BF of AD mice.

### Microglia SASP are identified by increased p16^INK4A^ and TNF-α expression, which promote chronic neuroinflammation and senescence in the BF of AD mice

To evaluate potential cellular senescence in the BF of AD mice, p16^INK4A^, a protein marker indicating irreversible cell-cycle arrest, was examined in brain sections from old 3xTgAD and WT mice (Fig. 7A–C). Population (%) of Iba1^+^ microglia expressing p16^INK4A^ increased in the BF of old 3xTgAD mice, which was significantly higher (39.48%) than that in younger AD mice (9.37%), *P* = 0.0007, also higher compared to age-matched WT mice (14.73%), *P* = 0.001, 2-tailed *t*-test with equal variance, *n* = 4–5 mice/group (Fig. 7D). Iba1^+^ microglia expressing p16^INK4A^ increased in old WT (14.73%) versus younger WT mice (3.44%) but not significantly. The average *R_p_* on colocalization of Iba1 and p16^INK4A^ within ROI of BF was significantly higher in old AD mice (*R_p_* = 0.58) versus in old WT mice (*R_p_* = 0.28) as well, *P* < 0.0001, and higher versus in younger AD mice (*R_p_* = 0.12), *P* < 0.0001 (Fig. 7E). This indicates the population of microglia with SASP (senescence-associated secretory phenotype) increased significantly within BF of old AD mice. Senescent microglia lose replication capacity for self-renewal. More significantly, by acquiring SASP they chronically secrete inflammatory cytokines, chemokines and toxic bioactive intermediates.^44^ SASP cells also mediate paracrine senescence to adjacent cells, thus amplifying and exacerbating inflammation within their local microenvironment.^45^ p16^INK4A^ expression was also observed in neuronal-like cells expressing human mutant tau protein in BF of AD mice (Fig. 7C and F), although their frequency (∼10%) was not as high as in Iba1^+^ microglia within the BF of AD mice. Another cellular senescence marker p21^cip1^, a cyclin-dependent kinase inhibitor, was also detected in Iba1^+^ microglia from brain sections from 3xTgAD mice (Supplementary Fig. 4), including in BF, subiculum, and hippocampus (with abundant deposition of amyloid ß plaques).

**Figure 8:**
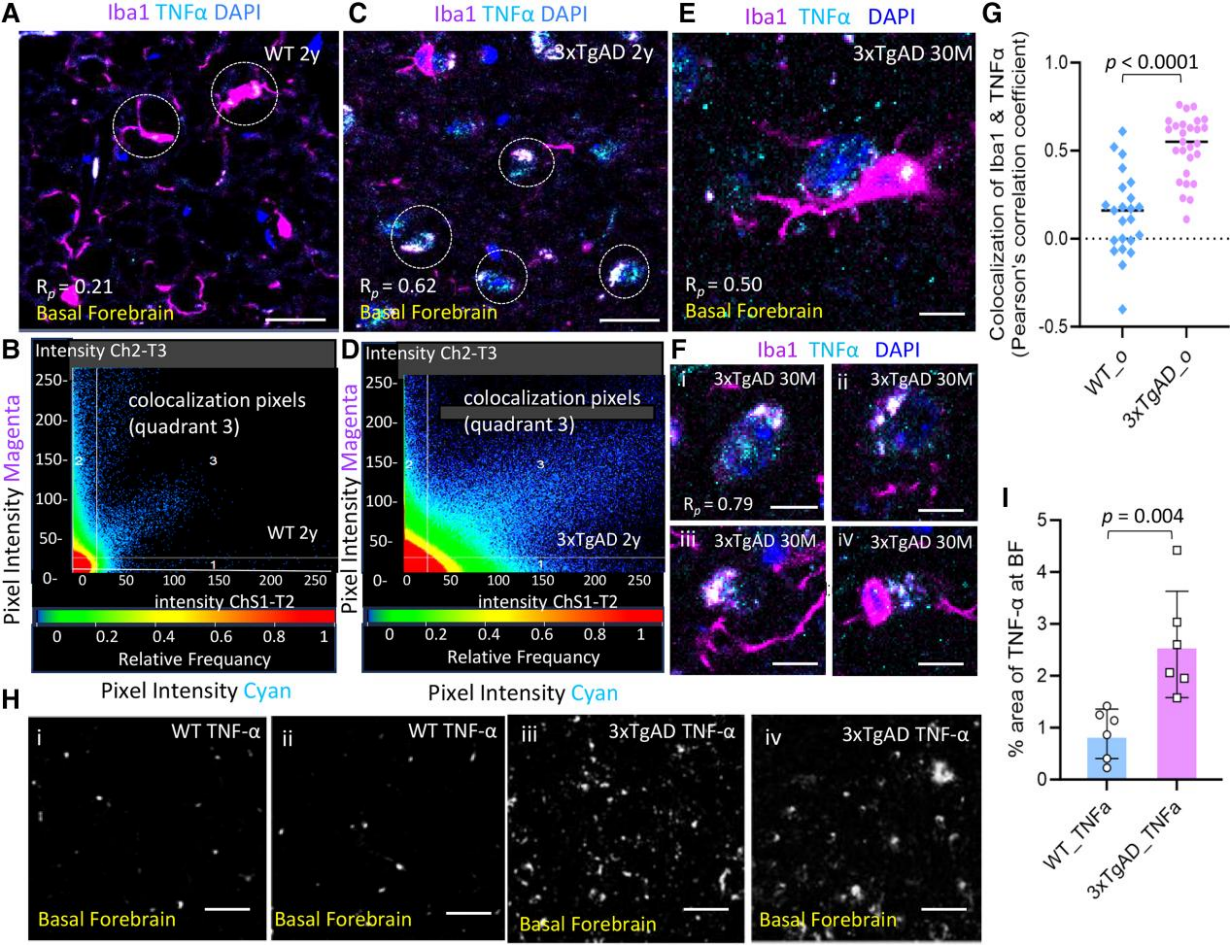
**Persistent increase of TNF-α in SASP microglia within BF in 3xTgAD mice.** Representative images showing the pro-inflammatory cytokine TNF-ɑ expressed in Iba1^+^ microglia and corresponding scatterplots on colocalization pixel intensity within BF of WT (**A–B**) and 3xTgAD (**C–D**) mice. (**E)** Representative image showed the persistent expression of TNF-ɑ in Iba1^+^ microglia from a very old 3xTgAD mouse (30 M), which spread to neighbouring cells and extracellular microenvironment (Fi–iv). (**F**) The Iba1^+^ microglia cells with TNF-α expression also displayed phenotype and deteriorating changes. **(G**) The average *R_p_* on colocalization of Iba1 with TNF-α was significantly higher in old 3xTgAD versus in old WT mice as evaluated from multiple brain sections. *P* < 0.001, unpaired 2-tailed *t*-test with equal variance, *t* = 6.584, *df* = 48; *F* = 1.761, *P* = 0.168; *n* = 3–5 mice/group. (**H–I**) The representative images revealed the area (%) with TNF-ɑ distribution at BF of WT (Hi–ii) and 3xTgAD (Hiii–iv) mice (16–24 months) by Image J analyses, *P*  *=* 0.004; 2-tailed *t*-test with equal variance, *t* = 3.73, *df* = 10; *F* = 4.607, *P* = 0.119; 2–3 sections/mouse, *n* = 4 mice/group. Scale bars, 50μm in **A** & **C**; 20μm in **E** & **F**; 100μm in H; *R_p_*, Pearson’s correlation coefficient.

Expression of the pro-inflammatory cytokine TNF-α was increased significantly in Iba1^+^ microglia within the BF of 3xTgAD mice (Fig. 8C–D) compared to age-matched WT mice (Fig. 8A–B). The persistence of expression of TNF-ɑ was also observed in Iba1^+^ microglia from very old 3xTgAD mice (30 M), which by spreading to neighbouring cells and the extracellular microenvironment potentially contributed to chronic inflammation, cellular senescence, and degeneration (Fig. 8E–F). The average *R_p_* on colocalization of Iba1 and TNF-α within BF was significantly higher in old AD mice versus in old WT mice as well (Fig. 8G), *P* < 0.0001, unpaired 2-tailed *t*-test with equal variance, *n* = 3–5 mice/group. The areas (%) showed TNF-α expression (Image J) was higher within BF of 3xTgAD compared to WT mice (Fig. 8H–I). *P* = 0.004, unpaired two-tailed *t*-test with equal variance, *n* = 4 mice/group.

**Figure 9:**
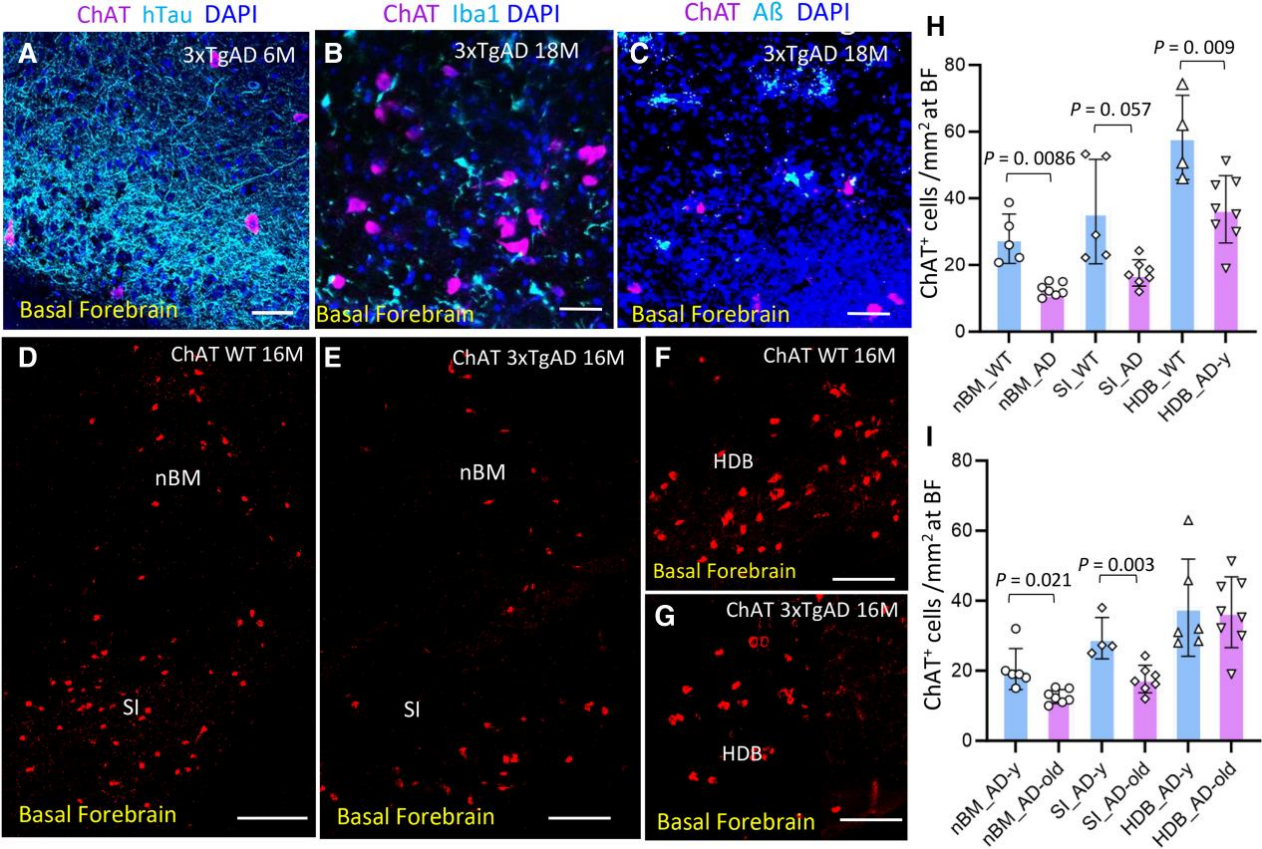
**Loss of ChAT^+^ cells within selected cholinergic nuclei at BF of AD mice.** Representative images indicate the microenvironment within BF of 3xTgAD mice. There were deposited hTau (ctan) fibrils (**A**), Iba1^+^ (ctan) microglia (**B**) and extracellular amyloid plaques (cran) at BF where the ChAT^+^ cells (magenta) located. (**C**) ChAT^+^ cell numbers were assessed at selected cholinergic nuclei (nBM, SI and HDB) within BF of 3xTgAD versus WT mice (*n* = 4–8 animal/group). Representative confocal tiling images showed the density of ChAT^+^ cell (red) numbers in the nBM and SI (**D–E**), and HDB (**F–G**) from WT and 3xTgAD mice (16 −24 M). (**H**) ChAT^+^ cell numbers in listed cholinergic nuclei of 3xTgAD versus WT mice. One-way ANOVA analysis: *F* = 11.61; *P* < 0.001. Unpaired 2-tailed *t*-test (Wetch’s correction for unequal variance) was performed between two groups. For nBM, *P* = 0.0086, *t* = 4.493, *df* = 4417; *F* = 13.80, *P* = 0.007; for SI, *P* = 0.057, *t* = 2.571, *df* = 4.362; *F* = 15.90, and *P* = 0.01; for HDB, *P* = 0.009, *t* = 3.213, *df* = 10; *F* = 1.563, and *P* = 0.563. (**I**) ChAT^+^ cell numbers in the nuclei of BF of younger versus old 3xTgAD mice. One-way ANOVA analysis: *F* = 11.61; *P* < 0.0001. Unpaired 2-tailed *t*-test between two age groups: for nBM, *P* = 0.021, *t* = 3.115, *df* = 5.98; *F* = 8.763, and *P* = 0.02 (Wetch’s correction). For SI, *P* = 0.003, *t* = 4.007, *df* = 9; *F* = 2.231. and *P* = 0.37; for HDB, *P* = 0.841, *t* = 0.205, *df* = 12; *F* = 1.879, and *P* = 0.433. Each data represents mean of ChAT^+^ cells at each region from sections of individual mouse. *n* = 5–8 mice/group. Scale bars, 100μm in panels **A**, **B**; 250μm in **C–E**; 200 μm in **F–G**. Abbreviations: nBM, nucleus Basalis of Meynert; SI, substantia innominate. HDB, nucleus of horizontal limb diagonal band of Broca.

Chronic neuroinflammation driven by accumulation of pathological tau and amyloid β protein and microglia activation and SASP creates a deleterious microenvironment within BF of AD mice (Fig. 9A–C), which exposes the vulnerable cholinergic system to neurodegeneration and loss.^46,47^ ChAT^+^ cell numbers were evaluated from multiple sagittal brain sections at BF from 3xTgAD and age-matched WT mice (2–4 sections/per animal, *n* = 4–7 animal/group) in several regions of cholinergic nuclei including nBM, SI and HDB. The boundaries of those cholinergic nuclei are defined on the mouse brain atlas^35^ (Supplementary Fig. 1). ChAT^+^ cell numbers were reduced significantly in the nBM and HDB of 3xTgAD mice (Fig. 9D–G, and H), and in the nBM and SI compared young with old 3xTgAD mice (Fig. 9I). The early loss of ChAT^+^ cells, and continued progressively with aging in AD mice, was associated with the cholinergic deficits and pathophysiology on neurovascular function seen here in neocortex rCBF haemodynamic responses to ACh in 3xTgAD mice.

**Figure 10:**
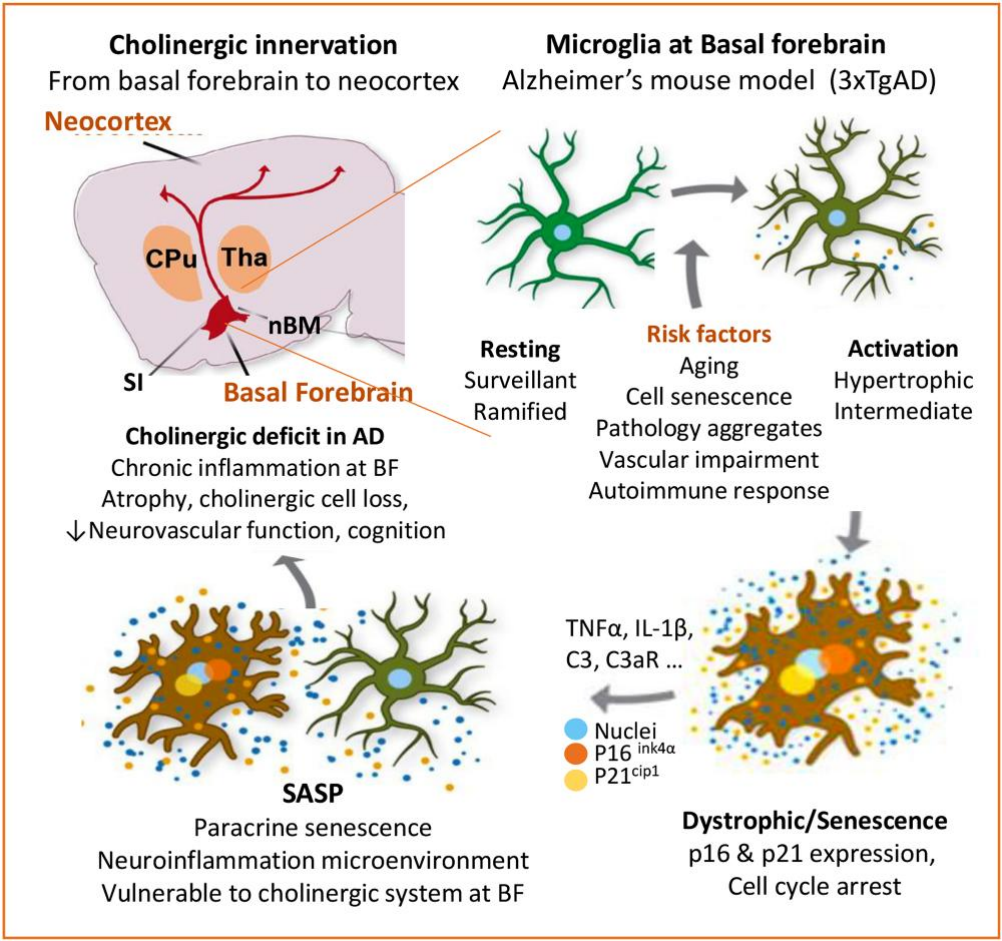
**Impact of chronic inflammation at BF on cholinergic deficits and rCBF hemodynamics in Alzheimer’s disease model.** Neocortex cholinergic innervation is derived from cholinergic nuclei within the basal BF, which modulate neurovascular function including CBF haemodynamic. In the brain of 3xTgAD model, aging-associated cellular senescence and the accumulation of pathological protein aggregates activate innate immunity at BF. Microglia cells, the major immune surveillance resident and effectors of innate immunity, become reactive and dystrophic/senescent, expressing a higher rate of irreversible cell-cycle arresting markers p16^Ink4a^ and p21^cip1^ and releasing pro-inflammatory cytokine TNF-α and other inflammation components with aging and disease progression. The acquired SASP (senescence-associated secretory phenotype) further amplified local inflammation and senescence, and reduced inflammation resolution. The chronic inflammation microenvironment is deleterious to the vulnerable cholinergic system at BF, contributing to cholinergic loss and cholinergic deficits on modulating neocortex rCBF hemodynamics to neuronal activity and cognition. Abbreviation: nBM, nucleus Basalis of Meynert; SI, substantia innominate; CPu, caudate putamen; Tha, thalamus; SASP, Senescence-associated secretory phenotype.

Schematic summary: Chronic inflammation at Basal Forebrain of Alzheimer’s brain linked to cholinergic deficits and altered rCBF haemodynamics. Neocortex cholinergic innervation is derived from cholinergic nuclei within BF. In AD brain, age-related cellular senescence and pathology protein aggregates activated innate immunity. Microglia cells, major immune surveillance residents and effectors of innate immunity become reactive and dystrophic/senescent. They release pro-inflammatory cytokines and active immune components, acquiring SASP and expressing p16^Ink4a^ and p21^cip1^. SASP-amplified chronic inflammation, paracrine senescence and reduced inflammation resolution, which is deleterious to the vulnerable cholinergic system at BF, contributes to cholinergic deficits in modulating rCBF haemodynamics in response to cognition in AD (Fig. 10).

## Discussion

### Altered neocortical rCBF haemodynamics to ACh is associated with cholinergic deficits in AD mouse model

The regional CBF haemodynamic response to local brain activity is critical for neurovascular coupling and cognitive function. Central cholinergic innervation is involved in modulating rCBF perfusion haemodynamics in response to neuronal activity.^10,16,17,23^ Previous studies have reported that patients with AD have reduced CBF perfusion in their posterior parietotemporal region as compared to age-matched controls, and central cholinergic stimulation produced a focal increase in rCBF in AD patients but not in controls.^18^ These findings suggest that enhancing cholinergic activity has the potential to improve rCBF perfusion in AD.^11,12^

In the current study, we found age-related differences in CBF haemodynamics in response to exogenous ACh administration (i.v.) in the neocortex region of 3xTgAD mice versus WT mice. The initiation and amplitude of % ΔrCBF to ACh challenge, as determined by responses of CBF haemodynamics from the basal level, were faster and higher in younger adult 3xTgAD mice (1y) than in age-matched WT mice (*P < 0.05), suggesting a deficiency of endogenous ACh levels with lower cholinergic tone and basal CBF perfusion level in vivo. Whereas cerebrovascular reactivity to external ACh administration was maintained, hence the % ΔCBF to ACh was higher as compensation in younger AD mice (Fig. 2H–I). The aetiology of the cholinergic deficits in AD brain remains uncertain, although it has been proposed that the selective retrograde atrophy in cholinergic nucleus basalis neurons is related to a compromise in cortical proNGF maturation.^48^ The % ΔrCBF response to ACh was significantly reduced in old AD (2y) versus in younger AD mice (Fig. 2E), which could involve both vascular aging- and disease-related components of cerebrovascular pathology. This may include amyloid angiopathy, decreased clearance of CNS ß-amyloid, tau-induced blood vessel abnormalities, and blood–brain barrier (BBB) impairment with disease progression.^30,38,49,50^ In this regard, the existing and/or exacerbated cholinergic deficit in aged AD mice limits the rCBF response reaching the highest rCBF response level (rCBF_max_) to neuronal activity from the lower basal rCBF level, even though the % ΔrCBF to ACh in old AD mice was not significantly lower compared to it in aged WT mice (Fig. 2C and I). As a consequence, cholinergic deficits could impair neurovascular coupling and lead to CBF hypoperfusion in brain, an important risk factor for dementia.

### Iba1^+^ microglia interact with tauopathy and exhibit a senescence phenotype in the BF of AD mice

Since neocortical cholinergic innervation derives from cholinergic projection neurons within the BF, such as from the nBM,^31^ the impact of AD pathology on the cholinergic system in the BF was evaluated in our study. Early deposition of tauopathy including mutant hTau and phospho-tau181 was evident within the BF of 3xTgAD mice, as previously reported in human AD.^51^ Our previous study confirmed that intracellular tau neurofibrillary tangles (NFT) containing mutant hTau and *p*Tau formed sarkosyl-insoluble aggregates in 3xTgAD mice.^33^ In current study, pathological tau protein was found to be coinciding at selected clusters of ChAT^+^ cells including the SI/nBM and HDB occasionally (Fig. 4), albeit the outcome and mechanism(s) accounting for how pathological tau could induce neurodegeneration and neuronal loss at BF of AD remain to be elucidated.

A prominent feature noted within the BF of AD mice was that Iba1^+^ microglia cells interact with tauopathy and display significant morphological changes. The diverse Iba1^+^ microglia morphology was evaluated by phenotyping and Fractal analysis, which demonstrated a reduction in ramified phenotype and an increase in dystrophic/senescent phenotypes (Fig. 6B–C). In contrast, in aged WT mice, dystrophic/senescent microglia were limited within the BF, suggesting those dystrophic/senescent microglia within the BF of AD mice were disease-associated.^52^ As microglia are the major surveillance and immune effector cells resident throughout brain, their interaction with tauopathy represents innate immunity activation in AD brain.^53^ Excessive and prolonged microglia activation, as well as other components of the innate and adaptive immune response, could drive chronic inflammation and be detrimental to the microenvironment and neighbouring cells, which, in turn, contribute to BF atrophy in individuals at risk of AD, as has been reported.^54^

### Microglia SASP drives persistent neuroinflammation at BF and potentially contributes to cholinergic loss in AD mice

In our study, increased expression of p16^INK4a^, an irreversible cell-cycle arrest marker (Fig. 7A and D), and TNF-α, a pivotal pro-inflammatory cytokine (Fig. 8C–E), were identified in Iba1^+^ microglia within the BF of AD mice indicating an acquired SASP (senescence-associated secretory phenotype). Another cellular senescence marker, p21^cip1^, was also detected in aged 3xTgAD mice (Supplementary Fig. 4). SASP cells possess a bioactive secretome to secrete numerous inflammatory cytokines, chemokines, reactive oxygen species, and other toxic bioactive intermediates.^44^ SASP microglia thus have the potential to promote an inflammatory microenvironment and propagate senescence to neighbouring cells via paracrine senescence;^45^ thus exacerbating local cells including cholinergic system degeneration at BF of AD. It has been suggested that SASP cells retain metabolic flexibility to generate sustained pro-inflammation cytokines and other bioactive factors,^55,56^ and they are largely resistant to apoptotic stimuli.^57^ As major immune surveillance cells in the brain, increased microglia SASP is a biomarker of immune senescence in AD brain, which could negatively impact their normal physiological functions that include promoting synaptic pruning, optimizing plasticity and debris clearance to maintain brain homeostasis.^58^ Microglia senescence reduced their capacity for inflammation resolution due to impaired phagocytosis; in such cases, pathological tau seeds engulfed within microglia are more readily spread within the AD brain; thereby, expanding the area with AD pathology and chronic inflammation.^59-61^

The molecular pathways involved in microglia-mediated inflammation to cholinergic loss is not well understood and will be further investigated. In our 3xTgAD model, the loss of ChAT^+^ cells across several cholinergic nuclei within the BF was indicated in middle-aged and old AD mice (Fig. 9). The accumulation of pathologic protein aggregates in BF such as tauopathy and amyloid ß aggregates are likely the targets of an immune response including ChAT^+^ cells colocalized with pathology Tau though seems not abundant. Tauopathy is a common feature in multiple neurodegenerative diseases besides AD, which is correlated with cognitive decline. It is notable that the neurotoxicity of intracellular tau aggregates (NFT) on neuronal survival remains somewhat controversial.^62,63^ Pathologic tau aggregation associated with cellular senescence has been reported in human AD and rodent models preceding neurodegeneration.^42,64,65^ The expression of p16^INK4a^ was also observed in cells bearing tauopathy within BF of AD mice in our study (Fig. 7C and F). Cellular senescence, chronic inflammation, and microglia-mediated synaptic elimination could play key roles in age-related neurodegeneration including cholinergic system in AD.^66-68^ In this regard, the clearance of senescent cells has been suggested to reduce chronic inflammation and cognitive decline.^65^ While brain cells bearing senescent markers including glia, neuronal cells, and vascular cells will increase in the population (%) with age advance (∼30% of microglia within BF of old AD mice, Fig. 7D). Senolytic agents potentially eliminate senescence cells, the long-term safety of this strategy and impact on brain cell volume, regeneration and cognition need more clarification. Alternatively, the activation of innate and adaptive immune responses could have a more directed impact on neuronal survival and are targets for intervention during disease progressing. Besides microglia phagocytosis and pro-inflammatory cytokines release, other components of immune responses, including autoimmunity, could be also important for preventing neuronal loss in the brains of Alzheimer’s disease.

#### Limitations and future directions

Our study supports the idea that chronic inflammation with cellular positive SASP microglia within BF is associated with cholinergic pathophysiology in the 3xTgAD mouse model. However, the use of the AD mouse to model AD in humans has its own limitations; even though the mouse model is driven by human transgenes associated with AD, the majority sporadic human AD may not necessarily be triggered and progress in the same manner. The initial trigger(s) of immune activation and resulting inflammation in BF of AD brain remain uncertain. There are other aetiology-related initiators that could, likewise, induce inflammation and impact neuronal loss in the complexity of human AD (sporadic and genetic AD cases). In addition to AD pathology, other potential factors such as the regional location of BF in the brain could make it more vulnerable or accessible to exposure to pathogens that are known to trigger the onset of neuroinflammation.^69^ The regional appearance and staging of pathological hallmarks may be more complex in the larger human AD brain. Furthermore, microglial cells (which are a major focus in our mouse AD study) are not the only players mediating inflammation in the brain, as other multiple components of innate and adaptive immune responses can also be involved in the process contributing to neurodegeneration.

Cholinergic projection neurons within the BF serve as the major cholinergic input to the neocortex and other brain regions including entorhinal cortex, hippocampus, amygdala and olfactory bulb.^4^ Cholinergic degeneration in BF precedes cortical pathology, and the atrophy of BF in AD is correlated to cognitive decline.^54,70,71^ A deeper understanding of AD aetiology that includes mechanisms causing the cholinergic deficit and neuronal loss are critical to aid the design and development of disease-modifying therapeutics. This includes defining potential targets involved in disease progression and when such targets may be responsive to therapy. Hence, our future studies will focus on mechanisms potentially underpinning neuronal degeneration/loss. Potential factors involved in disease initiating inflammation and disease progression, such as innate and adaptive immunity and the mechanisms that drive neurodegeneration, should be further investigated. Parallel neuropathology studies in human AD brain samples could be undertaken to evaluate translation. Prevention strategies such as vaccines and immune modulation could be successful in the design and development of disease-modifying therapy.

## Conclusion

AD is a common age-related neurodegenerative disease leading to dementia. The multiple risk factors proposed and incomplete understanding on aetiology of AD is linked to the poor success in the design and development of disease-modifying therapeutics. Age-related cerebrovascular diseases, cellular senescence, and chronic inflammation are major risk factors in pathogenesis of AD.^66-68,72^ Our studies revealed that microglia SASP accumulated with advancing age and disease progression within BF in an AD mouse model. This is initially associated with innate immune activation. Later, it becomes a biomarker of immune cell senescence,^73^ contributing to the unresolved chronic neuroinflammation and deterioration of the microenvironment of neighbouring cells. The accrual of cellular senescence is implicated across a diverse number of age-related diseases, including cancer and other age-related neurodegenerative disorders, albeit the consequences are quite likely different in these disorders.^73^ A previous study reported that a partial decrease in microglia did not influence tau pathology in aged mice,^74^ although clearance of senescent glial cells could prevent tau-dependent pathology and cognitive decline in a different study.^55^ Senescent cells are limited in normal aging in both human and mouse brains, which has several protective and cell survival properties as well, such as tumour suppression and limiting macrophage proliferation in atherosclerosis.^75,76^ The safe and long-term impact of senolytic agents on age-related neurodegeneration diseases and cognition remain to be further clarified.^77^ Illuminating the mechanism of chronic inflammation including the initial aetiology, impact of innate and adaptive immune response on pathogenesis and disease outcome, could provide a strategy to ameliorate neurodegeneration and neuronal loss in vulnerable brain regions of AD and related pathophysiology.

## Supplementary Material

fcae204_Supplementary_Data

## Data Availability

The data related to this study are available from the corresponding author upon reasonable request.
