# Supplementary material for: Chronic inflammation with microglia senescence at basal forebrain: impact on cholinergic deficit in Alzheimer’s brain haemodynamics
Source: Brain Commun. 2024 Jun 12;6(4):fcae204. doi: 10.1093/braincomms/fcae204 (PMC11228546; doi:10.1093/braincomms/fcae204)
Supplement: fcae204_Supplementary_Data [file fcae204_supplementary_data.pdf]

Supplementary material

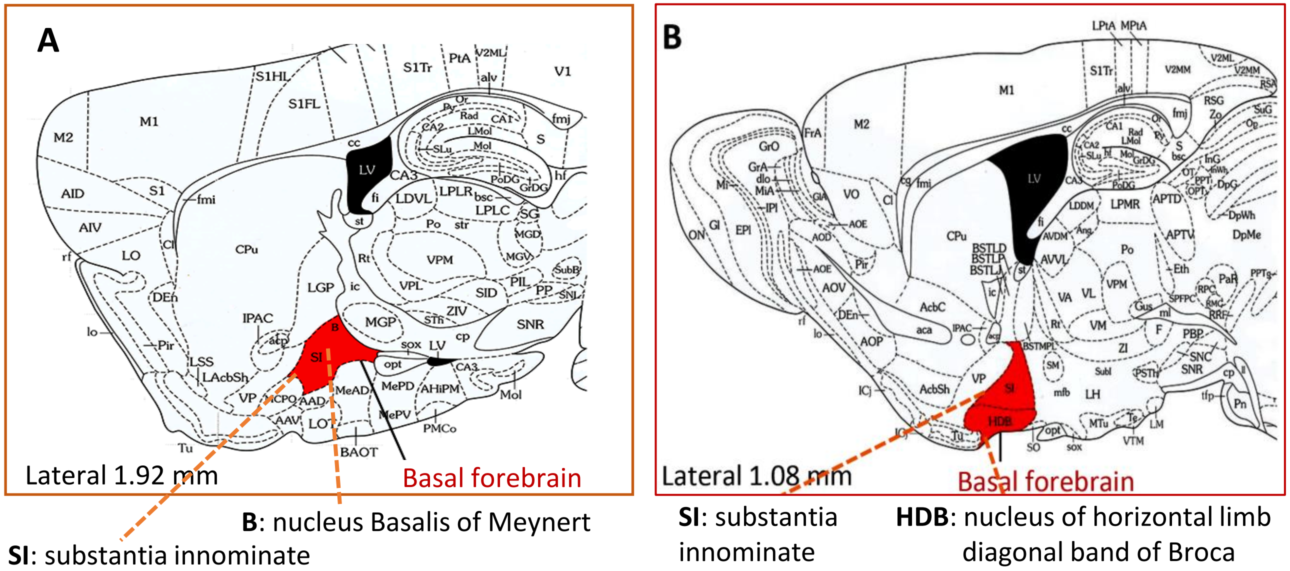

Supplementary Fig. 1 The basal forebrain (BF) regions investigated on mouse brain sagittal sections in this study. The BF regions are defined on the Mouse Brain Atlas (Paxinos and Frankline, 2001), and highlighted (orange) in Fig. 2A (B and SI) and 2B (SI and HDB). Abbreviation: **B**, Nucleus Basalis of Meynert (nBM); **SI**, Substantia innominate, **HDB**, Nucleus of horizontal limb diagonal band of Broca.

Source: Paxinos G and Franklin KBJ. The mouse brain in stereotaxic coordinates. Deluxe edition of the Atlas Academic Press 2nd edition. 2001

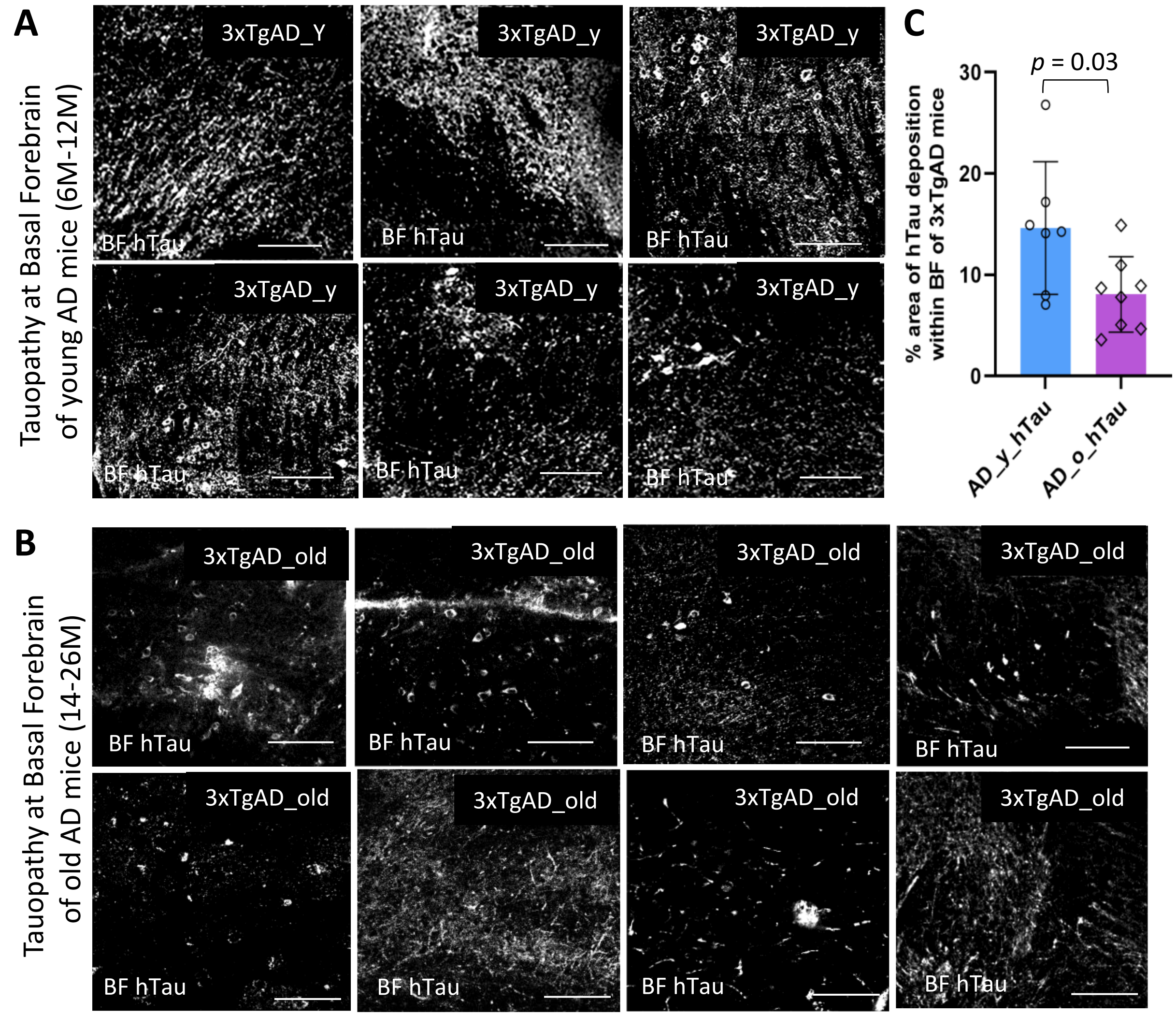

Supplementary Fig. 2 Age related deposition of tauopathy (P301L) in BF of young and old 3xTgAD mice. **(A)** Representative images revealed deposition of hTau at BF on sagittal sections from young AD mice (6 - 12M). (**B**) Representative images revealed hTau deposition at BF on sagittal sections of old AD mice (16 - 26M). (**C**) The % area with hTau deposition within BF was quantified from multiple brain sections of young and old 3xTgAD mice. $p = 0.03$; two tailed t –test; t=2.428, df = 13; F = 3.095, P = 0.165; n = 4-6/group. Scale bar = 100 µm. BF, Basal forebrain, hTau, human Tau.

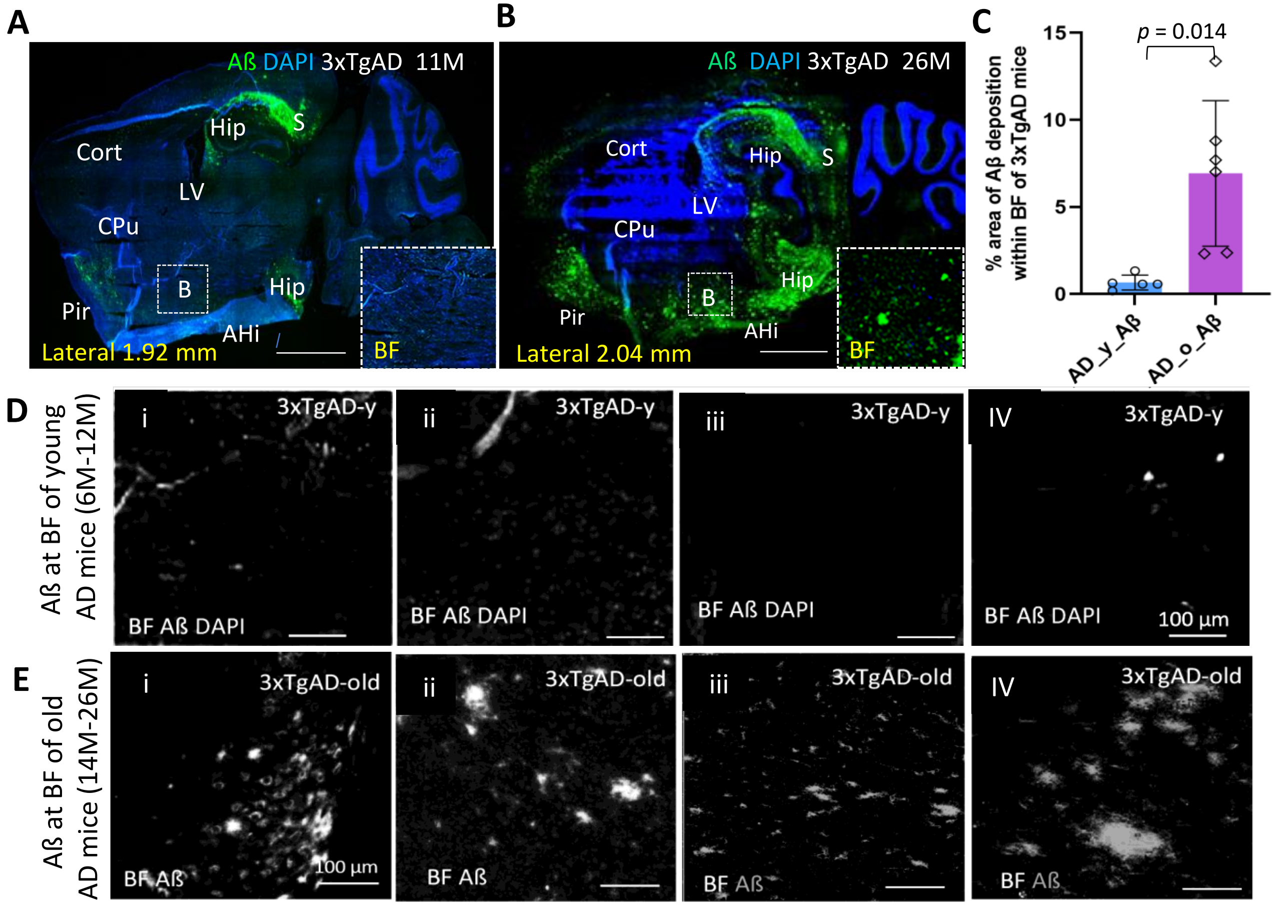

Supplementary Fig. 3 Age related deposition of amyloid ß within BF of 3xTgAD. (A) Representative images revealed amyloid ß deposition (green) on a sagittal brain section of a young AD mouse (11M). Amyloid ß plaques appeared at subiculum of hippocampus hub, piriform cortex and AHi, but not evident at basal forebrain (BF) at this stage. (**B**) Representative image revealed abundant deposition of amyloid ß plaques in an old 3xTgAD mouse (26M) including BF. (**C**) The % area of amyloid ß deposition at BF is evaluated from multiple young and old 3xTgAD mice. $p = 0.014$, unpaired two-tailed t-test with Welch's correction for unequal variances; $t = 3.648$, $df = 5.12$; $F = 99.53$, $P < 0.001$; $n = 3\text{-}5$ mice/group. (**D-E**) Representative images on amyloid ß deposition (white) at BF from multiple young or old 3xTgAD mice . Abbreviations: B, Basal Forebrain; Cort, cortex; Pir, piriform cortex; LV, lateral ventricle; S, subiculum; AHi, amygdalohippocampal area; CPu, caudate putamen. Scale bar: 750 µm in A-B, 100 µm in D-E.

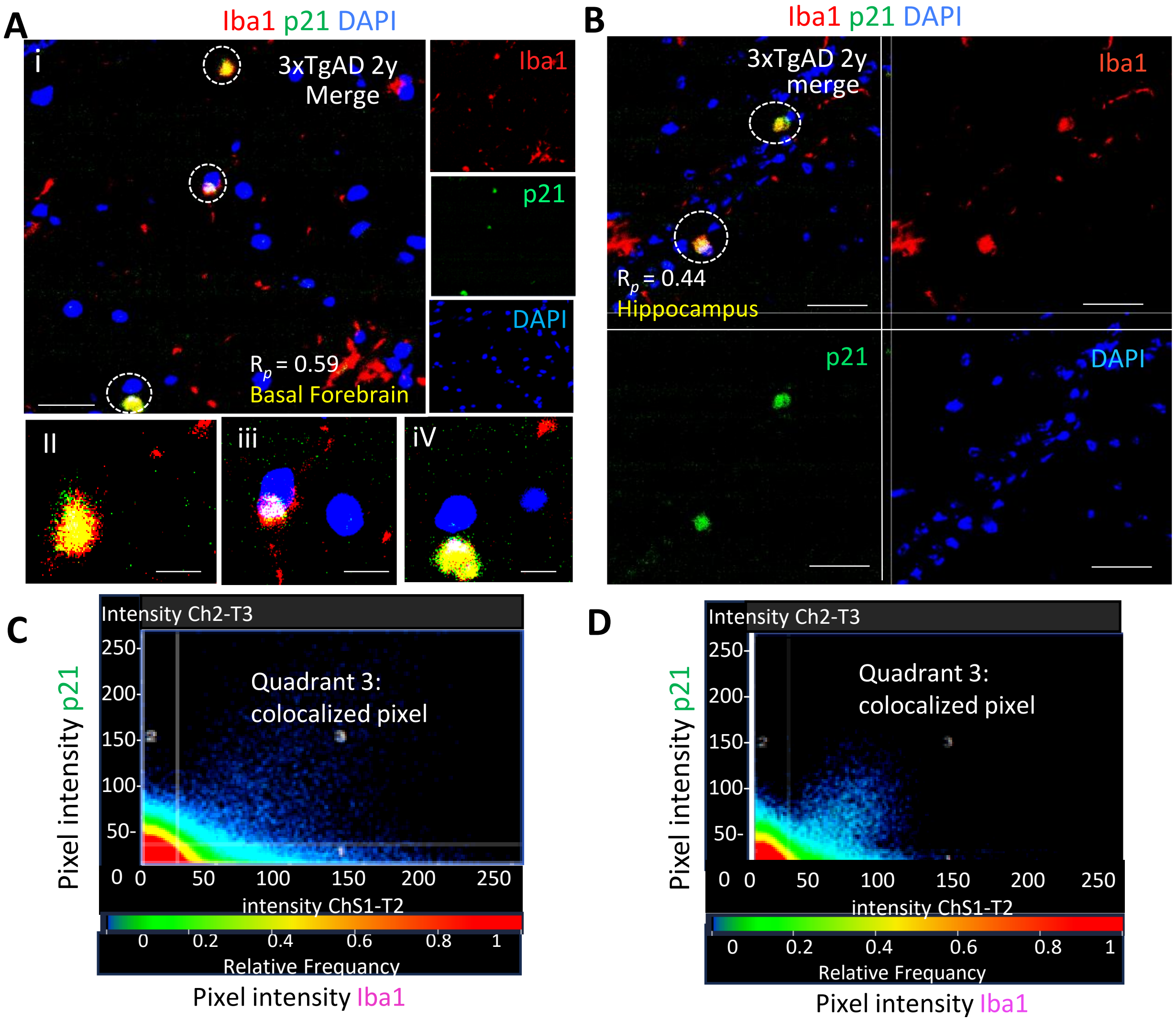

Supplementary Fig. 4 Senescence marker p21^{cip1} detected in microglia at 3xTgAD mouse brains. **A** (**i**) Representative immunofluorescence images revealed p21^{cip1} expression (green) collocated in Iba1^{+} microglia (red) at basal forebrain of a 3xTgAD mouse (2y), R_p = 0.59. (**ii-iv**) Images showing p21^{cip1} colocalized in Iba1^{+} microglia (yellow) showing dystrophic/senescence phenotype. **B** Representative images showing p21^{cip1} expression (green) colocalized in Iba1^{+} microglia (red) at hippocampal hub in an old AD mouse, R_p = 0.44. **C-D** Scatterplot of Iba1 and p21 pixel intensity on image A and B and colocalized pixels distributed in Quadrant 3. $R_{p\,:}$ Pearson's correlation ecoefficiency. Scale bare = 100 µm in A & B, and 10 µm in A, b-c.

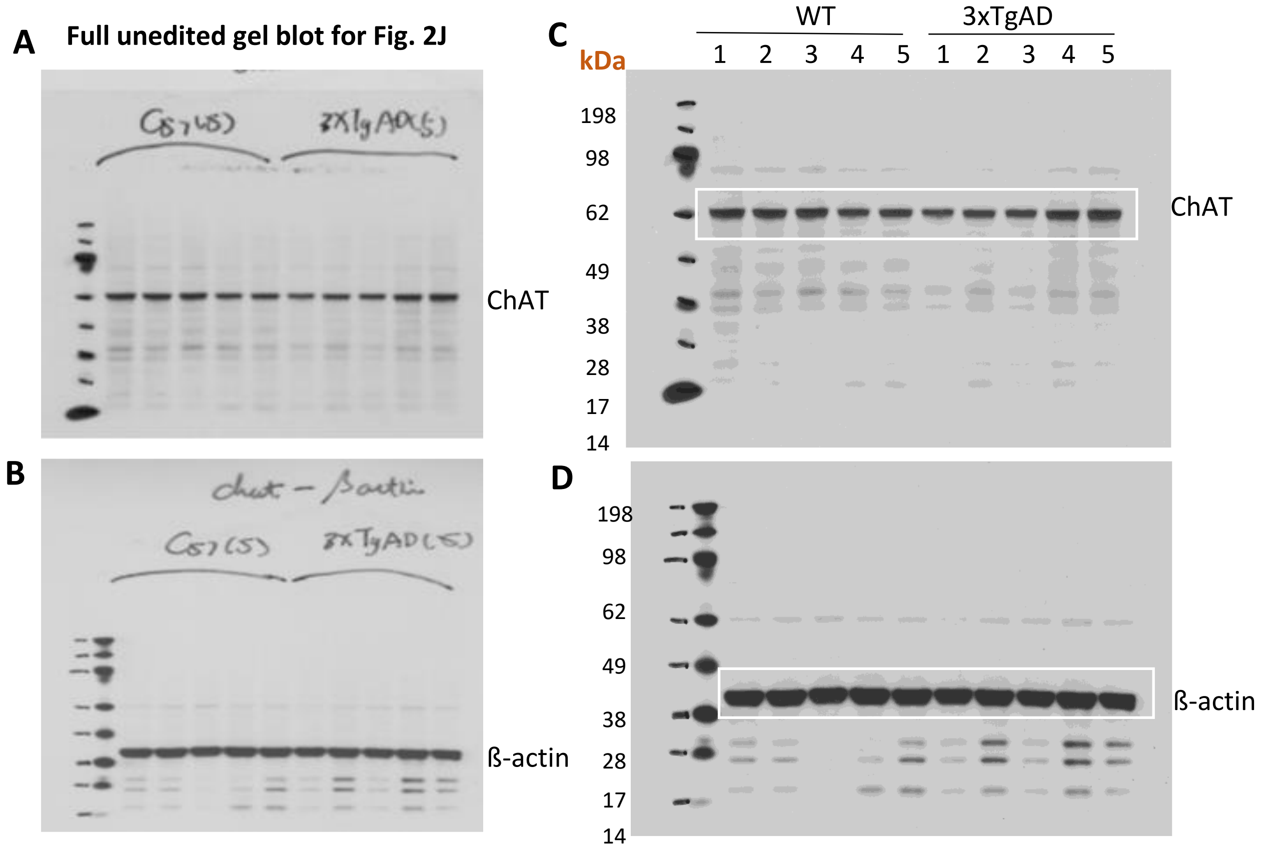

Supplementary Fig. 5 **(A-B)** Unedited images of western gel blots on ChAT and β-actin protein levels loaded with protein samples from neocortex of WT and 3xTgAD mice (1y) in the same blot. *n* = 5 animals/group. (**C-D**) The protein bands of ChAT and β-actin from the same blot were selected (within white lines) for **Fig. 2J.** The density of protein bands were quantified by image J, and the density of ChAT protein bands in each line were normalized with correlated band density of β-actin from the same blots (**Fig. 2K**).
